# Isotopic Depletion Increases the Spatial Resolution of FPOP Top-Down Mass Spectrometry Analysis

**DOI:** 10.1021/acs.analchem.3c03759

**Published:** 2024-01-16

**Authors:** Marek Polák, Jiří Černý, Petr Novák

**Affiliations:** § Institute of Microbiology of the Czech Academy of Sciences, 14220 Prague, Czech Republic; ‡ Department of Biochemistry, Faculty of Science, Charles University, 12843 Prague, Czech Republic; † Laboratory of Structural Bioinformatics of Proteins, Institute of Biotechnology of the Czech Academy of Sciences, 14220 Prague, Czech Republic

## Abstract

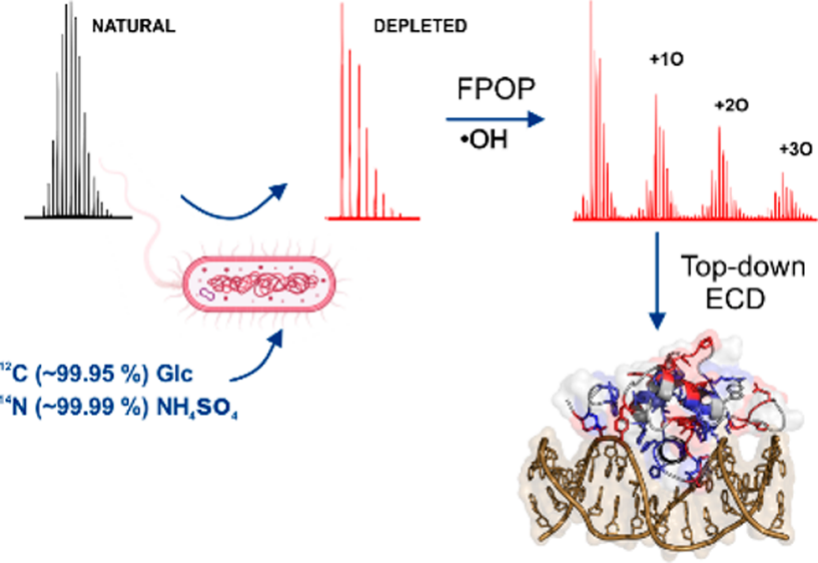

Protein radical labeling, like fast photochemical oxidation of proteins (FPOP), coupled to a top-down mass spectrometry (MS) analysis offers an alternative analytical method for probing protein structure or protein interaction with other biomolecules, for instance, proteins and DNA. However, with the increasing mass of studied analytes, the MS/MS spectra become complex and exhibit a low signal-to-noise ratio. Nevertheless, these difficulties may be overcome by protein isotope depletion. Thus, we aimed to use protein isotope depletion to analyze FPOP-oxidized samples by top-down MS analysis. For this purpose, we prepared isotopically natural (IN) and depleted (ID) forms of the FOXO4 DNA binding domain (FOXO4-DBD) and studied the protein–DNA interaction interface with double-stranded DNA, the insulin response element (IRE), after exposing the complex to hydroxyl radicals. As shown by comparing tandem mass spectra of natural and depleted proteins, the ID form increased the signal-to-noise ratio of useful fragment ions, thereby enhancing the sequence coverage by more than 19%. This improvement in the detection of fragment ions enabled us to detect 22 more oxidized residues in the ID samples than in the IN sample. Moreover, less common modifications were detected in the ID sample, including the formation of ketones and lysine carbonylation. Given the higher quality of ID top-down MSMS data set, these results provide more detailed information on the complex formation between transcription factors and DNA-response elements. Therefore, our study highlights the benefits of isotopic depletion for quantitative top-down proteomics. Data are available via ProteomeXchange with the identifier PXD044447.

Structural proteomics has considerably advanced the field of structural and molecular biology in recent years by enabling us to address questions related to the structure and dynamics of proteins and their complexes. Methods of structural proteomics are particularly useful for studying transcription factor–DNA interactions^1^ such as those involved in transcription. Transcription is primarily regulated by transcription factors (TFs), proteins that specifically activate or inhibit this process by forming complexes with DNA. For instance, Forkhead transcription factor “O” 4 (FOXO4) is known to bind core motif 5′-(C/A)(C/C)AAA(C/T)A-3′ (Insulin Response Element) and further activate transcription that includes energy metabolism control.^2,3^ Yet, despite extensive research in this area, our understanding of transcription regulation and interaction between TFs and their binding motifs remains limited. To better understand these processes, we must further clarify the structural mechanism underlying the formation and interaction of protein–DNA complexes.^4−6^

Protein–DNA complexes have been studied using cross-linking reactive probes,^7−9^ hydrogen–deuterium exchange (HDX)^7,8,10−12^ and radical covalent labeling.^1^ It was demonstrated that surface mapping of biomolecules, detected by high-resolution MS analysis, offers useful information related to the protein structure or its interaction with other biomolecules.^1^

Various radical probes are currently available, with diverse reactivity toward different amino acids,^13−18^ but the most commonly used labeling chemistry consists of modification by hydroxyl radicals.^19^ Among the methods involving hydroxyl radicals, the most promising approach was introduced by Hambly and Gross in 2005 and is referred to as fast photochemical oxidation of proteins (FPOP).^20^

In FPOP, proteins are irreversibly labeled in a quench-flow capillary system by a single hit of hydroxyl radical, generated from hydrogen peroxide by an excimer laser. This reaction is immediately quenched by a suitable quenching reagent in the flow system. The rationale of this approach lies in the preferential oxidation of solvent-accessible side chains of the investigated molecule, and thus mapping of the protein structure.^21,22^ Hydroxyl radicals promote modification of 14 of 20 naturally occurring amino acids. The most reactive are the sulfur-containing amino acids, namely, cysteine and methionine, followed by the residues of the aromatic amino acids, namely, phenylalanine, tyrosine, histidine, and tryptophan.^23−26^ As such, FPOP has been applied to various studies, including mapping of protein conformational changes, protein–protein interactions,^27^ the structure and topology of membrane proteins,^28−31^ and large biomolecules^32^ such as antibodies.^20,27−31,33,34^

Although bottom-up MS analysis has long been a method of choice for studying FPOP modified samples, top-down protocols have recently demonstrated their potential for analyzing singly oxidized proteoforms.^1,35,36^ Originally, a top-down technology was introduced to determine not only biomolecule sequences but also post-translational and other (bio)chemical modifications of biomolecules.^37−39^ One of the advantages of analyzing samples by top-down MS is the precise determination of the molecular mass of proteins and other proteoforms. However, when the molecular mass of the analyzed species exceeds ∼1 kDa, the monoisotopic peak ceases to be the most abundant signal in the spectrum of the analyzed protein or peptide. Mass spectra of multiply charged proteins and fragment ions over ∼10 kDa do not produce observable monoisotopic peaks anymore, so the ion signal is dispersed into several, commonly overlapping isotopic peaks. As a result, the MS/MS spectra are complex and show a low signal-to-noise ratio (SNR).^40,41^

Precisely designed to overcome these difficulties, the technique of protein isotope depletion was introduced in 1997.^42^ Protein isotope depletion relies on incubating bacteria in media with depleted heavy isotopes, e.g., carbon and nitrogen. Several studies have described the benefits of isotope depletion for analyzing proteins up to ∼20 kDa,^43−45^ but Gallagher et al. stood out for using protein isotope depletion to improve the resolution of MS/MS spectra and thus fragment ion assignment.^46^ More recently, Popovic et al. applied protein depletion to analyze the proteome of cells by 21T-FT-ICR mass spectrometry.^47^ In common, these studies leveraged the potential of protein depletion for improving the accuracy of mass determination and sequence coverage of biomolecules by MS.

Considering the above, this study aimed at exploring the concept of protein isotope depletion and its advantages for increasing the spatial resolution of top-down MS analysis of FPOP samples. To this end, we prepared an isotopically depleted version of FOXO4-DBD and oxidized this sample under two different conditions, that is, with and without its DNA binding element, IRE. First, we used a bottom-up MS approach to analyze isotopically natural (IN) protein samples to acquire single-amino acid information. Subsequently, we applied a top-down MS approach to analyze an isotopically depleted (ID) version of the protein, which resulted in enhanced sequence coverage and more precise assignment of labeled residues. ID fragmentation yielded new ions in the MS/MS spectra, which were not detected in the IN spectra. The additional fragment ions offered more detailed information about the solvent-accessible FOXO4-DBD surface, thus enabling *ab initio* design of a FOXO4-IRE structural model.

## Experimental Section

### Materials and Chemicals

All solvents (LC/MS grade) and chemicals were purchased from Merck (Germany) unless stated otherwise.

#### Protein Expression of Isotopically Natural (IN) FOXO4-DBD

The expression was performed in Terrific-Broth (TB) medium. All details about the expression of IN-FOXO4-DBD can be found in the Supporting Information.

#### Protein Expression of Isotopically Depleted (ID) FOXO4-DBD

The expression of FOXO4-DBD was performed in M9 minimal medium containing glucose (99.95% of ^12^C, Merck) and ammonium sulfate (NH_4_SO_4_, 99.99% of ^14^N, Merck) as a source of carbon and nitrogen, respectively. All details about the expression of ID-FOXO4-DBD can be found in the Supporting Information.

#### Protein Purification

Both IN- and ID-FOXO4-DBD were purified in the same fashion, as described in detail in the Supporting Information.

### FOXO4·IRE Complex Formation

Forward (5′-GAC TAT CAA AAC AAC GC-3′) and reverse (5′-GCG TTG TTT TGA TAG TC-3′) complementary strands, whose sequence was retrieved from a previous study,^19^ were obtained from IDT (Coralville, USA) in an HPLC quality. A stock solution of dsIRE was prepared by mixing both strands in an equimolar ratio in LC-MS water. The mixture was incubated at 90 °C for 3 min and cooled to room temperature to form dsIRE. The 30 μM and 35 μM samples of IN/ID FOXO4-DBD and dsIRE, respectively, were mixed to form a complex and ensure that all protein was bonded in complex with DNA. The complex was diluted in 150 mM ammonium acetate, pH 6.8, and incubated at room temperature for 15 min to obtain IN/ID-FOXO4·IRE complex at 30 μM final concentration.

### Fast Photochemical Oxidation of Proteins (FPOP)

The FPOP labeling was performed in an in-house built quench-flow reactor as described previously.^1^ Prior to the FPOP experiment, glutamine (10 mM) was added to the protein samples. Briefly, ID/IN FOXO4-DBD (30 μM) with and without dsIRE (35 μM), in 150 mM ammonium acetate, pH 6.8, was continuously mixed in a T-mixer with H_2_O_2_ (15 mM during reaction). The mixture was irradiated by an excimer laser (COMPex 50 KrF, Coherent Inc., USA). A mixture of the sample and H_2_O_2_ was subjected to a single shot at a wavelength of 248 nm, frequency 15 Hz, energy 107 mJ, 20 ns pulse duration, and 2.24 mJ/cm^2^ radiant exposure. The exclusion volume was 16%. The reaction was quenched by immediate mixing with 75 mM methionine. The samples were collected in an Eppendorf tube containing 3000 U of Catalase (Merck, USA).

### Top-Down MS Detection

Protein–DNA samples were denatured by adding 4 M urea and 1 μM MgCl_2_; the mixture was incubated at a bench for 15 min. DNA was digested by adding 1 μL of benzonase endonuclease (Merck) and incubated at 30 °C for an additional 15 min. The mixture was then loaded onto a reverse-phase microtrap column (Optimize technologies, USA), desalted using 0.1% FA, and eluted with 80% ACN, 0.1% FA. The desalted protein was further diluted 5 times using 30% ACN, 0.1% FA solution, and sprayed using a nESI source in positive mode at 120 °C desolvation temperature. MS and MS/MS analyses were performed on a solariX XR mass spectrometer equipped with 15 T magnet (Bruker Daltonics, Billerica, USA), which was externally calibrated using the sodium trifluoroacetate to achieve 1 ppm mass accuracy. The time-of-flight was set to 1.1 ms, with the collision energy ranging between −3.0 to −5.0 V, and data were acquired with 2 M data point transient starting at 200 amu. At first, MS intact spectra were acquired in a broadband mode (*m*/*z* 200–2500) by accumulating ions for 0.1 s–0.2 s and collecting 128 scans.

For MS/MS, singly oxidized protein ions of three charge states (+14, +13, +12) were isolated using a multiCASI (multicontinuous accumulation of selected ions) in a quadrupole and then transferred to ICR cell for electron-capture dissociation (ECD). The single oxidized ions of IN-FOXO4 were isolated at 844.50, 909.30, and 984.90 amu, and the isolation window was ±1.0 amu. ID-FOXO4-DBD singly oxidized ions were isolated at nominal values 843.93, 908.77, and 984.42 amu, and the isolation window was ±0.6 amu. An ion-accumulation from 3.0 to 5.0 s was tuned to reach the intensity of the precursor ion image current of ∼10^8^ prior to the ECD experiment. The ECD was done by setting the parameters to obtain optimal fragmentation as follows: ECD pulse length 0.065–0.075 s, bias 0.90–1.0 V, and lens 14.0–15.0 V. The hollow cathode current was 1.5 A. Control spectra of unmodified ions were acquired using the same condition as the oxidized ones. Data were acquired by collecting 128 scans in a technical triplicate for both apo and holo forms.

### Top-Down Data Processing

Raw data were processed using Data Analysis 5.3 (Bruker Daltonics, Billerica, USA), MS2Links software,^27^ and in-house built software. Details related to top-down data processing are included in the Supporting Information.

### Bottom-Up LCMS Detection

Samples for bottom-up analysis were digested using Trypsin/LysC (Promega, USA) and LysC (Promega, USA). Respective protease was added at a protease:protein ratio 1:40 (m:m) and incubated overnight at 37 °C. Additional protease (m/m 1:20) was added after overnight incubation for another 6 h. IRE was digested by adding bensonase endonuclease (250 U, Merck) to the sample for 30 min at 37 °C. Digestion was terminated by addition of trifluoroacetic acid (TFA, 0.1%).

LC was performed to separate the peptides as described previously,^1^ albeit with one minor modification. An LC run consisted of a 35 min linear gradient of 2–35% solvent B. LC was directly hyphenated to a trapped ion mobility-quadrupole time-of-flight mass spectrometer (timsTOF Pro, Bruker Daltonics) for MS/MS analysis. MS/MS analysis was performed as described previously.^1^

LC-MS analysis was performed on a timsTOF Pro mass spectrometer. MS analysis was performed using the same method as MSMS, but without collisional dissociation and fragment accumulation.

Data were processed using a PeaksX+ Software (Bioinformatic Solutions Inc., Waterloo, ON, Canada) against a FOXO4-DBD sequence as described previously.^1,48^ Peptide intensities were extracted from LC-MS trace using Data Analysis 5.3 (Bruker Daltonics, USA) for all observed charge states, quantified, and statistically analyzed, as described previously.^49^ The mass spectrometry proteomics data have been deposited to the ProteomeXchange Consortium via the PRIDE^50^ partner repository with the data set identifier PXD044447.

### Homology Modeling

The homology model of the FOXO4/IRE complex was based on the available crystal structure 3l2c,^51^ extending the 3l2c template DNA sequence “–CTATGTAAACAAC–” to the IRE “GACTATCAAAACAACGC” sequence. The necessary nucleotide substitutions as well as residues missing at the 5′ and 3′ termini were modeled using the MMB program.^52,53^ The backbone conformation of the newly built termini was set to the canonical B form DNA using dinucleotide conformation class (NtC) BB00.^54,55^ The geometry of the initial model was further equilibrated during a 200 ns molecular dynamics simulation in GROMACS 2021.4^56^ using the ff14SB^57^ force field for FOXO4 and the tumuc1 force field^58^ for DNA. The model was placed in a rectangular box with the 10 Å shortest distance from the walls. The box was filled with TIP3P model water, and Na^+^ and Cl^–^ ions were added to reach a charge-neutral system with 100 mM salt concentration. The system was simulated with noncovalent cutoffs of 10 Å at 300 K and 1 bar with the V-rescale modified Berendsen thermostat and the Parrinello–Rahman barostat.

## Results and Discussion

Top-down MS analysis of FPOP enables us to determine the protein–DNA interaction interface between FOXO4 and dsDNA (namely DAF16), as shown in our recent study.^1^ However, the complex spectra and lower sequence coverage prevent us from reaching single-amino acid resolution, as in the bottom-up MS approach. To increase fragment intensity and to reduce the complexity of top-down MS spectra, we prepared IN and ^13^C/^15^N-doubly depleted (ID) versions of FOXO4-DBD to investigate the benefit of isotopic depletion for studying the interaction interface between FOXO4 and IRE by top-down MS analysis. We therefore expressed and purified the DNA-binding domain of FOXO4 at a length of 82–186^3^ (numbering according to the FOXO4 wild-type sequence) containing two (G^–2^S^–1^) additional residues located in the N-terminus. So as to simplify the top-down MS data interpretation, we further used the 1 to 107 common numbering of *c* and *z* fragment ions. The conversion of fragment ion numbering to the wild-type FOXO4 sequence is shown in Figure S1.

**Figure 1:**
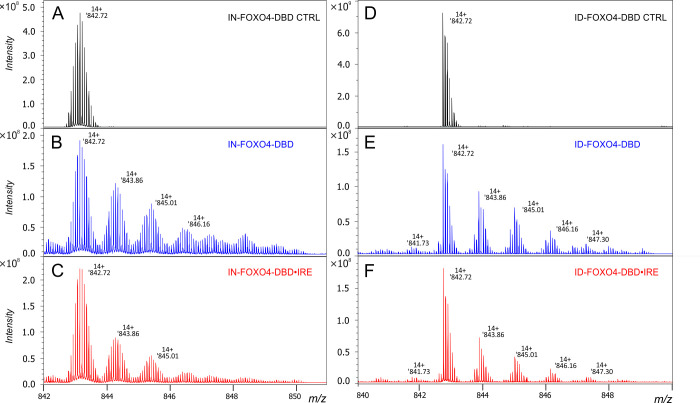
Zoomed mass spectrum on a +14-charge state (*m*/*z* 842–851) showing an isotopic distribution of isotopically natural (IN-, A) and isotopically depleted (ID-, D) FOXO4-DBD. Fast photochemical oxidation of IN-FOXO4 without (B) and with (C) dsIRE. Fast photochemical oxidation of ID-FOXO4 without (E) and with (F) dsIRE.

Initially, TB and M9 media were used to recombinantly express IN and ID protein, respectively (Methods in the Supporting Information). Both proteins were purified by using the same protocol. The intact protein analysis revealed the homogeneity of both protein samples (Figure S2) and confirmed the isotopic depletion of FOXO4-DBD (Figure 1A,D), while electrophoretic mobility shift assay (EMSA, Methods in the Supporting Information) demonstrated the ability of both proteins to bind dsIRE (Figure S3). Subsequently, the ID and IN forms of FOXO4-DBD were oxidized with and without dsIRE using FPOP. Figure 1B,C shows multiple oxidized proteoforms of IN-FOXO4-DBD with and without dsIRE, respectively, after FPOP. Similarly, Figure 1E,F displays multiple oxidized proteoforms of ID-FOXO4-DBD with and without dsIRE, respectively. With dsIRE (Figure 1C,F), the proteoforms were less oxidized than in solution alone (Figure 1B,E). These results confirm the protection of residues directly or indirectly involved in the protein–DNA interaction.

**Figure 2:**
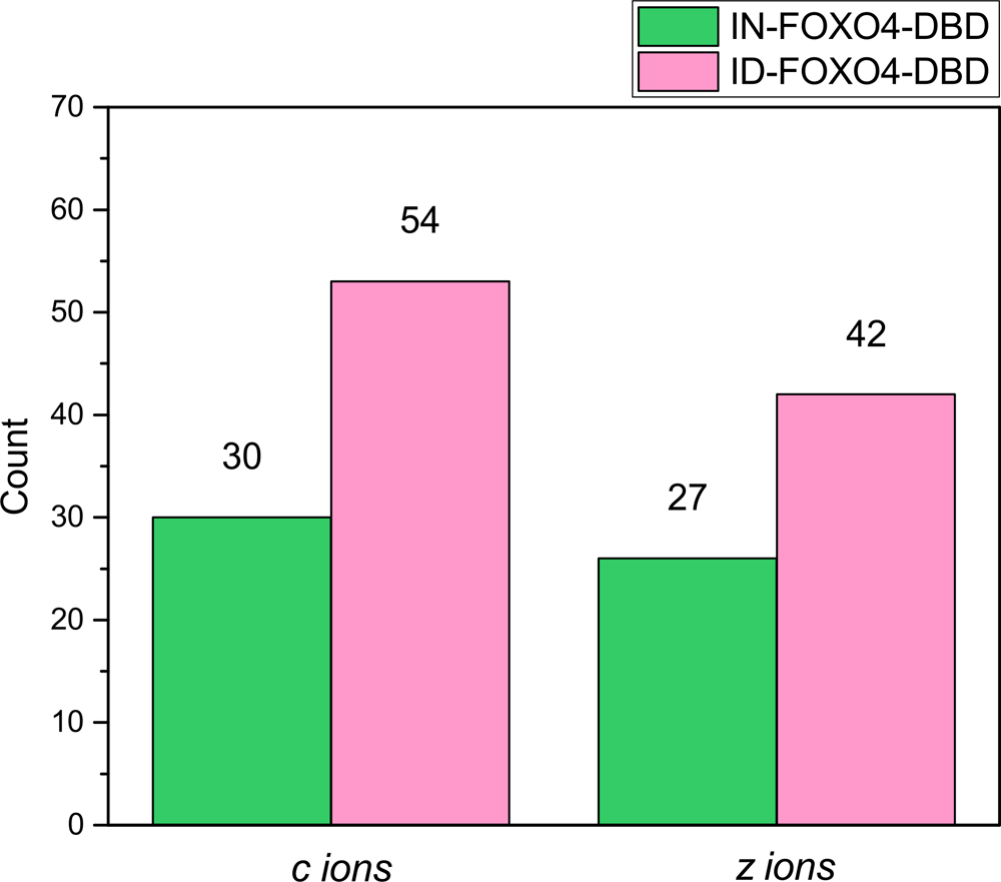
Histograms displaying the number of quantified fragment ions generated by ECD fragmentation of singly oxidized precursor ions of IN-FOXO4-DBD and ID-FOXO4-DBD.

Given the confirmation of the interaction between FOXO4-DBD and IRE by EMSA (Figure S3) and by intact MS analysis (Figure 1B,C,E,F), the investigation proceeded with top-down MS analysis. In order to demonstrate the advantage of isotopic depletion for analyzing singly oxidized proteins, singly oxidized proteoforms of IN and ID samples were isolated in the quadrupole and fragmented by ECD in the ICR cell (Figure S4).^35^ By combining multiCASI simultaneous isolation of three charge states with ECD fragmentation, we were able to detect 101 nonoxidized fragment ions (57 *c* ions, 44 *z* ions) when analyzing IN samples. However, only 57 fragment ions (30 *c* ions, 27 *z* ions; see Figure S5A) were intense enough for quantification, resulting in a sequence coverage of 27% (see Figure S5B). Under the same experimental conditions, three charge states were isolated and fragmented using the ID sample. The isotopic purity of the ID protein allowed us to isolate oxidized ions in the quadrupole with a narrower isolation window of ±1 and ±0.6 Da for IN and ID samples, respectively. In total, 148 fragment ions were then annotated and manually validated, including small ions, *c*3, *c*4, and *z*3, which did not show other observable isotopic peaks (Figures S6 and S7). Unlike in the IN sample, 95 fragment ions (54 *c* ions, 42 *z* ions; see FigureS8A) were intense enough for quantification, resulting in a sequence coverage of 45% (Figure S8B). Thus, another 24 *c*-ions and 15 *z*-ions were available for quantification in ID (Figure 2) compared to in IN samples.

**Figure 3:**
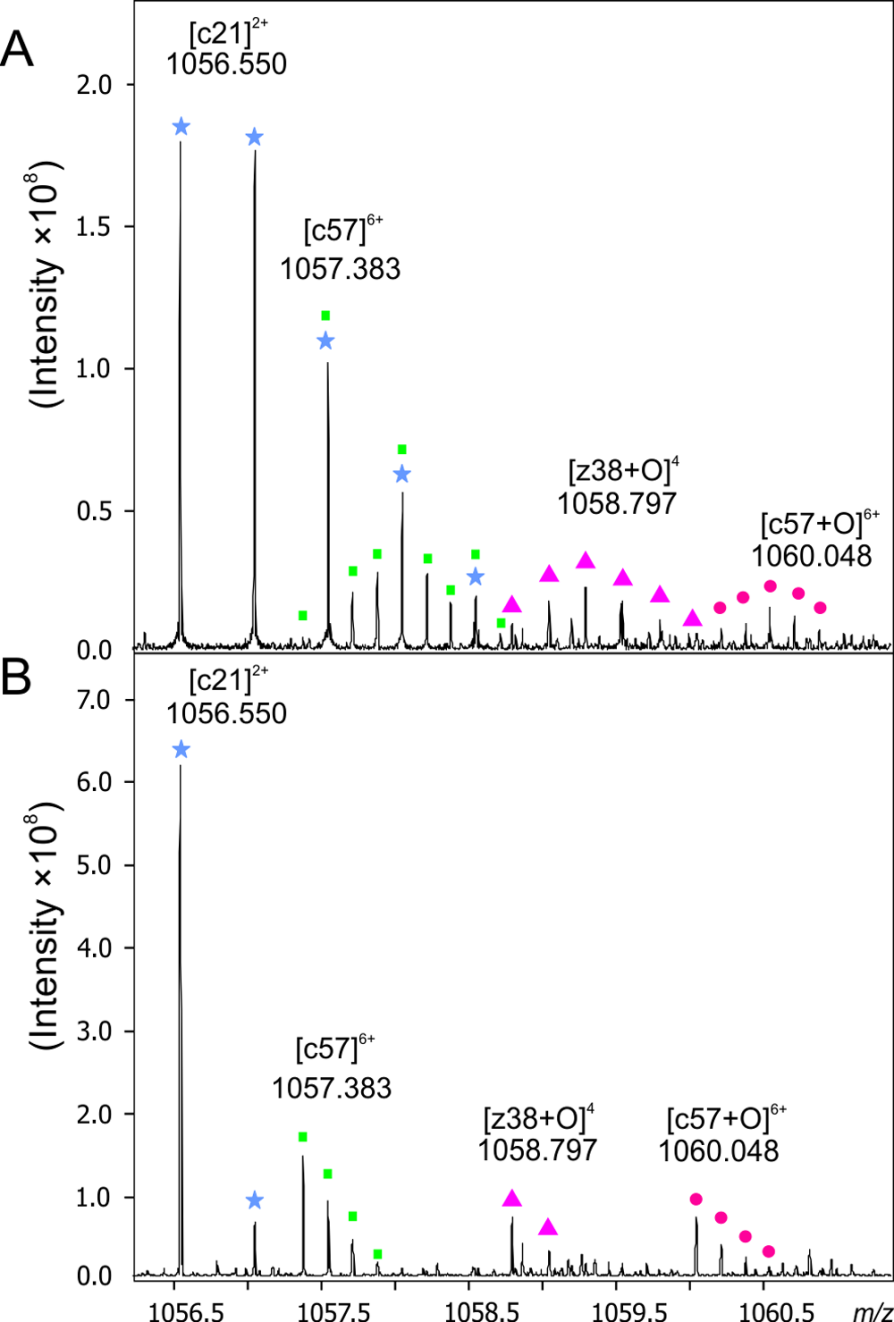
Zoomed ECD spectrum obtained upon fragmentation of isotopically natural (A) and isotopically depleted (B) FOXO4-DBD. The [c21]^2+^ is indicated with blue asterisks; the low-abundance [c57]^6+^ fragment ion is denoted by green squares; and its oxidized form, [c57+O]^6+^, is indicated by pink dots. The oxidized fragment ion [z38+O]^4^ is denoted by magenta triangles.

ID MS/MS spectra displayed (i) lower complexity than IN spectra, which greatly reduced the overlap of isotopes/fragment ions, (ii) a monoisotopic peak for all fragment ions, and (iii) an increased signal-to-noise ratio (SNR) (Figure S9). To further illustrate these improvements, we can consider the *m*/*z* region 1056–1061, which shows the difference in the complexity of the ECD spectra between IN and ID oxidized proteins (Figure 3).

Figure 3A clearly shows the overlap of three isotopes for two fragment ions, [c21]^2+^ and [c57]^6+^, thus precluding reliable quantification of the [c57]^6+^ fragment ion. Moreover, its oxidized form, [c57+O]^6+^, has a very low signal-to-noise ratio, which is close to the limit of detection. In contrast, fragment ion intensity is approximately three times higher in ID (Figure 3B) than in IN MS/MS spectra. Hence, the [c21]^2+^ fragment ion consists of only two peaks, and both [c57]^6+^ [c57+O]^6+^ fragments are now observed as intense ions in spectra, where the most abundant peak is the monoisotopic peak, which significantly exceeds the noise level.

During FPOP, the high reactivity of ·OH radicals promotes not only oxygen additions (+15.9949 Da) but also other modifications (Table S1).^59^ For instance, the conversion to the keto form^60^ (addition of +13.9793 Da) or lysine carbonylation^36,61^ (loss of −1.0313 Da) can also be detected during the FPOP analysis. In a typical bottom-up data analysis,^62^ where small peptides are created by enzymatic digestion and subsequently separated on a reversed-phase column, these modifications are easily detected by DDA analysis. However, these modifications are difficult to detect by top-down MS analysis for several reasons: (i) they are not major products of the reaction and thus are not observed at a higher intensity, and (ii) they may overlap with other reaction products in the MS/MS spectrum.^1,35^

**Figure 4:**
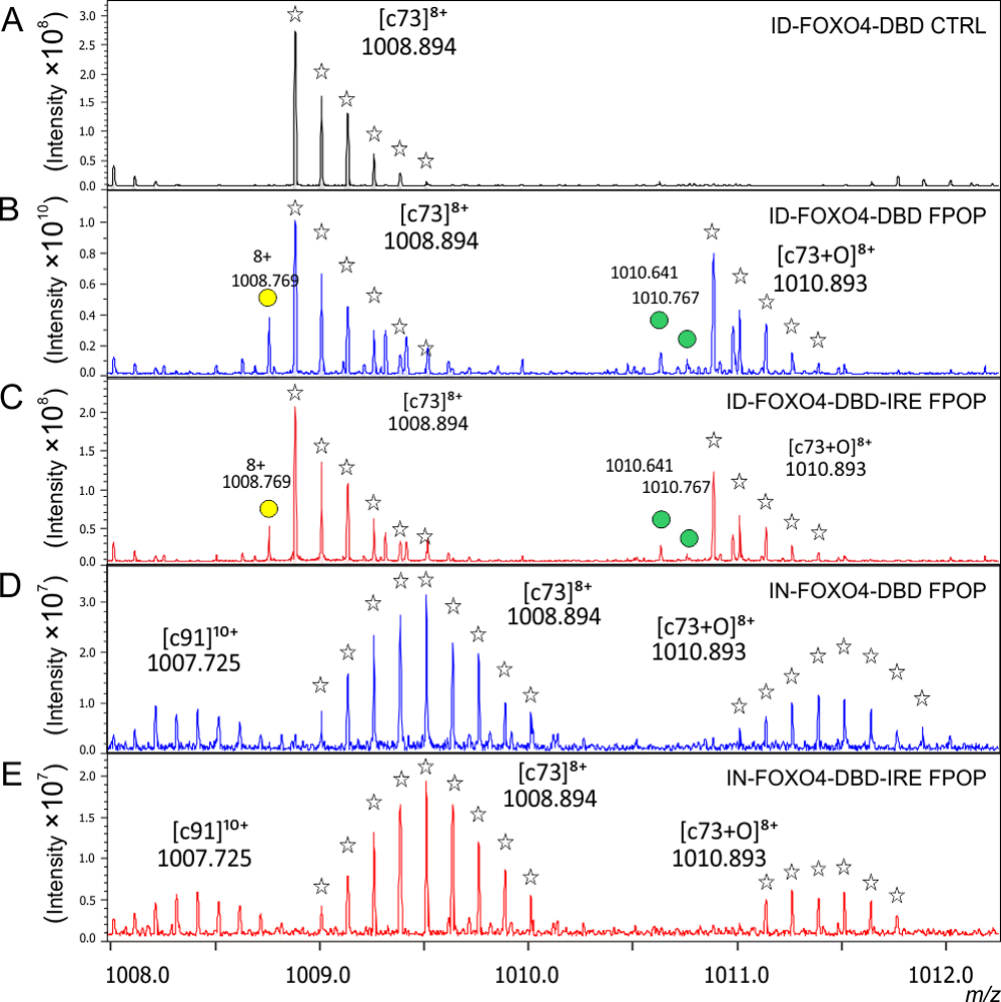
MS/MS spectrum zoomed in the *m*/*z* range 1008–1012.300. The control ECD spectrum of unmodified ID-FOXO4-DBD is colored in black in the top panel (A). The ECD spectrum of oxidized ID-FOXO4-DBD with (B) and without IRE (C) is colored in blue and in red. The isotopic distribution of both [c73]^8+^ and [c73+O]^8+^fragment ions is denoted by transparent asterisks. Yellow dots denote lysine carbonylation within the protein, represented by the loss of 1.013 Da, while the green dots represent the oxidation of protein to its keto form (+13.9793). An ECD MS/MS spectrum of IN-FOXO4-DBD without IRE (D) and with IRE (E) shows no visible lysine carbonylation or oxidation to keto form.

By simplifying the mass spectra and improving the signal-to-noise ratio (Figure S9), isotopic depletion helps to overcome the aforementioned limitations. As a result, these modifications can be detected in top-down MS/MS spectra of ID samples, as exemplified by the [c73]^8+^ fragment ion (Figure 4). The control spectrum does not show any oxidized [c73+O]^8+^ fragment ion (Figure 4A, black). But an MS/MS spectrum of oxidized FOXO4-DBD without (Figure 4B,D; blue) and with the IRE (Figure 4C,E; red) provided an oxidized [c73+O]^8+^ fragment ion. Additionally, oxidation to a keto form (indicated by green dots) and protein carbonylation (indicated by a yellow dot) were also detected during MS analysis, albeit to a lesser extent with IRE. This result confirms both the wide reactivity of ·OH radicals toward different residues and the protection of some residues by IRE (Figure 4B,C). Panels D and E of Figure 4, conversely, both show that neither lysine carbonylation nor keto-oxidation is identified in the zoomed-in view of the MS/MS spectrum of IN-FOXO4-DBD.

**Figure 5:**
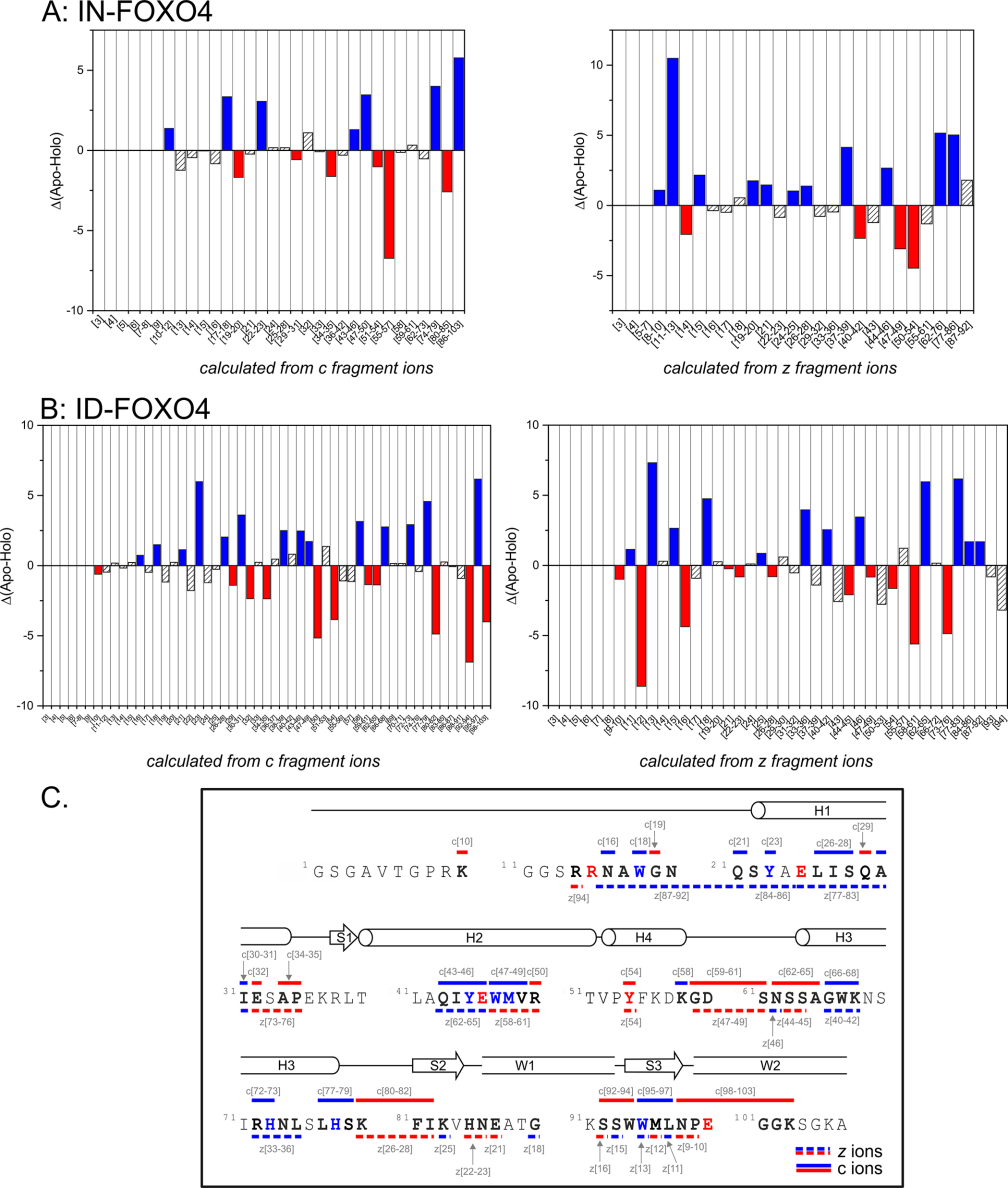
Plots indicating changes in oxidation rates between apo and holo forms of isotopically natural FOXO4 (A) and isotopically depleted FOXO4 (B); assessed by ECD fragmentation in multiCASI mode (Figure S5, Figure S8). Blue histograms represent changes in which region/residue was protected by IRE, and red histograms represent changes which resulted in deprotection of region/residue by IRE. (C) Changes obtained in ID-FOXO4-DBD were visualized into the differential oxidation map of FOXO4-DBD. The bold sequence represents spatial resolution achieved by fragmentation of isotopically depleted FOXO4-DBD. Colored residues were also detected by bottom-up analysis, as shown in Figure S11B and Table S1.

**Figure 6:**
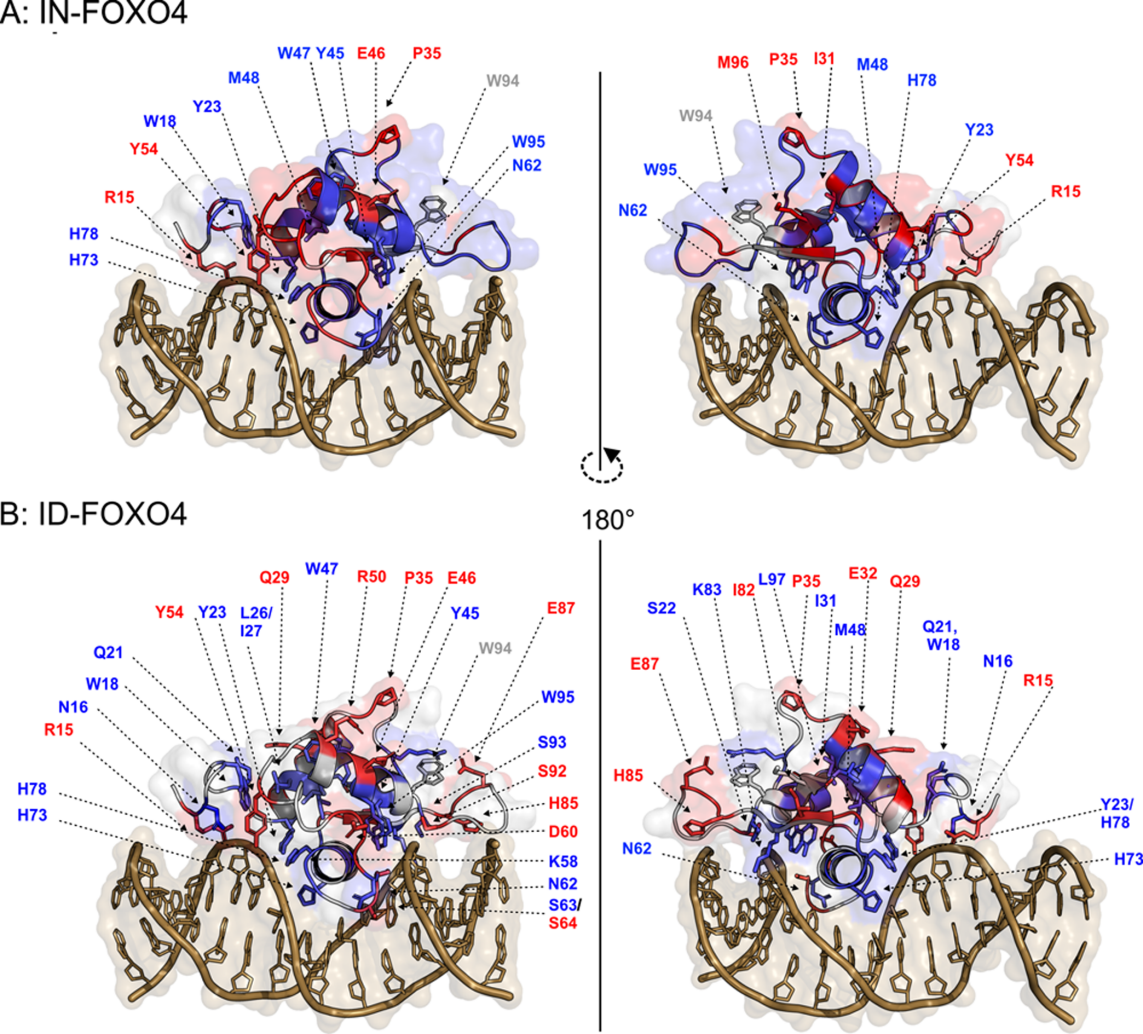
An *in silico* structural model of FOXO4-DBD·IRE (PDB template 3L2C)^22^ with the highlighted differently oxidized regions/residues detected by both top-down analyses for natural version (A) or depleted version (B) of FOXO4-DBD. The individual residues detected in either bottom-up approach or deduced from top-down were highlighted in the model and colored. Blue: regions/residues detected as more modified in apo form; red: regions/residues detected as more modified in holo form.

To obtain structural information based on the assignment of oxidized residues and the increasing intensity of fragment ions, the extent of oxidation was calculated for both the apo and holo forms. This allowed us to visualize the differences between vicinal fragment regions (Figure 5). The difference map for isotopically natural FOXO4 represents an example of lower sequence coverage (Figure 5A). In this case, only several residues might be assigned as oxidized based solely on a sequence coverage and on amino acid reactivity toward hydroxyl radicals. Thus, the overall extent of oxidation is a combination of the sum of extents of oxidations of residues located in each region and their exposure toward solvent. In contrast, fragmenting isotopically depleted protein provides increased signal-to-noise ratio, which resulted in elevated sequence coverage, and thus, more residues might be assigned as oxidized ones (Figure 5B). Analysis of the ID protein revealed changes in the oxidation patterns of several residues of FOXO4-DBD upon binding to IRE (Figure 5C). These were further visualized in an *in silico* model of FOXO4-IRE (Figure 6, Figure S10). The first detected oxidized residues, K10, N16 and W18, were protected upon the complex formation and deduced from c[10], c[16], and c[18] fragment ions. Residues N16 and W18 were found to be directly interacting with the IRE according to the structural model (Figure 6) and also according to the previous study.^51^ This is also consistent with the z[87–92] region. Helix H1 (S22-A34) contains several residues that were protected by IRE, in particular residues Q21, Y23, L26/I27, and I31, which were covered by c[21], c[23], c[26–28], c[30–31], z[77–83], and z[84–86]. This is in agreement with the previously published HDX^8^ and structural studies.^51,63^ However, we have also observed a higher oxidation rate for Q29, E32, and P35 deduced from c[29], c[32], c[34–35], and z[73–76], located throughout the H1 helix and intervening loop far away from the protein–DNA interface (Figure 6, Figure S10). Residues in helices H2 and H4, namely, Y45, W47/M48, and Y54,^51^ showed different oxidation patterns, with some being less oxidized and some being more oxidized upon protein binding to IRE. The residues were covered by regions c[43–46], c[47–49], z[62–65], c[54], and z[54]. Moreover, R50, oriented toward the solvent,^64^ was found to be more oxidized in the presence of IRE (Figure 5C, Figure 6).^1,51^ The protection of K58 and deprotection of D60 residue was deduced based on regions c[58], c[59–61], and z[47–49]. Oxidation of N62 is deduced from z[46], alongside the region c[62–65]/z[44–45] which pinpoints the oxidation to both S63 and S64 residues. One may hypothesize that both N62 and S63 are protected upon the complex formation,^3,51^ while S64 residue is oriented more toward the solvent and thus might be more oxidized upon complex formation (Figure 6).^51^ A helix H3 (G66-H78), the main contributor of interaction with the major groove of IRE,^51^ was found to be heavily protected. Residues of helix H3, namely, W67, H73, and H78, are covered by regions c[66–68], c[72–73], c[77–79], z[33–36], and z[40–42] and display the direct protection of helix by the major groove of IRE. Next, there has been a multitude of residues oxidized on strand S2 and wing W1 (F81–S93) bearing different oxidation patterns. Residues K83 and S93, covered by z[25] and z[15], were both protected. This observations are supported by a structural model and complementary mutagenesis studies^3^ demonstrating direct interaction of K83 and S93 residues with the DNA. Even though F81 is located next to I82 and also is more reactive, we pinpoint hotspot to the I82, because residue F81 is located away from the solvent and interacts with IRE in our model. Thus, residues I82, H85, E87, and S92, covered by regions z[26–28], z[22–23], z[21], and z[16], were detected as more oxidized.

Finally, data from isotopically depleted samples allowed us to resolve the solvent accessibility of residues located at strand S3 at a single residue resolution. Residues W94, W95, and M96 are covered by regions z[12], z[13], z[14], c[92–94], and c[95–97]. Overall effect of IRE binding is the stabilization of strand S3 and deprotection and protection of W94 and W95 residues, respectively, as W94 interacts with IRE.^3,51,63^ The analysis of smaller *z* ions led to the assignment of L97 and P99 as oxidized ones based on [z8] and [z10] fragment ions, respectively. Altogether, the isotopic depletion and selective gas-phase enrichment and fragmentation of oxidized ions lead to the detection of 30 residues, namely K10, N16, W18, Q21, Y23, L26/I27, Q29, I31, E32, P35, Y45, R50, K58, D60, N62, S63, S64, H73, H78, I82, K83, H85, E87, S92, S93, W94, W95, M96, L97, and P99. Out of them, 22 residues were deduced solely from the isotopically depleted top-down data set. The other 7 were also detected using the bottom-up analysis described below.

### Bottom-Up Analysis

To assess the top-down MS data, the same isotopically natural samples were also subjected to a bottom-up analysis. The LysC and Trypsin/LysC digestion of IN samples yielded 22 peptides, providing a sequence coverage of over 96% (see Figure S11A). Across the sequence of FOXO4-DBD, 18 residues were modified (Figure S11B and Table S1). Nine residues, namely, W18, Y23, Y45, W47, M48, H73, H78, W94^#^, and W95, showed decreased oxidation in the complex with IRE, indicating protection. By contrast, residues R15, E46, Y54, W94, W94^##^, and E100 showed increased oxidation/modification levels upon complex formation. Five residues were not affected by IRE binding. The single-residue resolution of the bottom-up approach supported data from our *ab initio* model, in line with previously published mutagenesis studies,^3^ which reported that most residues involved in the interaction with the major groove of DNA were less oxidized/modified.

The agreement between the bottom-up and top-down results demonstrates the usefulness of the top-down and complementarity of both techniques. For instance, residues W18, Y23, Y45, Y54, H73, H78, W94, W95, and M96 differed in the extent of their modification upon IRE binding in the bottom-up data set, as observed by top-down MS analysis. This confirms that top-down MS analysis is a reliable technique for detecting oxidation levels at different residues.

Notwithstanding the above, some differences were detected between the results of the two techniques: (i) Bottom-up workflow benefits from LC for resolving even isobaric/isomeric modifications prior to mass spectrometric detection, a feature that has been referred to as “sub amino acid resolution”.^34^ As a case in point, oxidized W94 can have several forms depending on its position on the indole ring. This finding was also detected by the top-down workflow, albeit only as a cumulative modification, represented as a sum of individual extents and without indicating the exact position of the modification (Figure S12). (ii) Limited sequence coverage, especially for larger proteins, is a known weakness of top-down techniques in general. Thus, residue oxidation determined by bottom-up can be overlooked by top-down approaches due to the lack of usable fragment ions intense enough for detection, as in the case of W47/M48 residues, both of which were detected in bottom-up but not individually in the top-down spectra. (iii) Bottom-up analysis of all proteoforms present in the sample also detects modifications other than the +16 Da^22^ due to oxidation resulting from enzymatic digestion, as shown by R15/R72 deguanidylation, E25, E46, and E100 decarboxylation, and H73 and H78 conversion into aspartate in our bottom-up data set (Figure S11, Table S1). In the top-down experiment, we performed targeted gas-phase accumulation of species with mass increased by +16 Da (indicative of oxidative modification) prior to fragmentation. For this reason, all other modifications were not identified in the top-down experiment. Nevertheless, this limitation could be easily circumvented by including these other theoretical modifications in the accumulation, if necessary. Despite requiring the identification of the species to be included, selective gas-phase accumulation is a great advantage of top-down analysis for significantly enriching the included forms.

In our experiments, this enrichment enabled the detection and assignment of 22 new oxidized residues that were not detected by bottom-up MS analysis, as discussed above. These oxidations were impossible to detect by a bottom-up workflow given the low limit of detection, which is especially limiting for forms with low levels of oxidation and concentrations in the mixture of trypsinated peptides. As such, the benefits of top-down MS analysis in improving the limit of detection can actually outweigh its other limitations.

One obstacle to the broader use of isotopic depletion in MS analysis is the availability of processing software. The current software portfolio is restricted to the natural occurring isotopic distribution, and the data deconvolution mainly relies on averagine function.^45^ This function does not allow the deconvolution of an altered isotopic pattern. Nevertheless, this problem may also be solved in the near future by adopting ion deconvolution using measured collision cross sections of trapped ions.^65^ For larger proteins, MSMS spectra are necessarily complex, regardless of using protein isotope depletion. However, for small and midsized proteins,^41^ the advantage of isotopic depletion is significant.

## Conclusion

Protein isotope depletion improves the detection and quantification of FPOP oxidation by a top-down MS analysis. This approach has three main advantages: (i) more precisely isolating singly oxidized ions in quadrupole filter prior to fragmentation; (ii) enhancing the intensities of unmodified and oxidized fragment ions; and (iii) improving the resolution of fragment ions in MS/MS spectra by reducing the number of isotopic peaks and thus reducing the overlap of existing peaks, including those of individual isotopes.

Combining isotopic depletion with top-down analysis of FPOP samples boosts sequence coverage by 19% and identifies 22 more oxidized residues in comparison to bottom-up MS analysis. Nevertheless, bottom-up and top-down analyses show highly consistent results, demonstrating their complementarity. The more detailed information on the interaction interface obtained in this study enables the *ab initio* design of FOXO4·IRE complex formation. Even beyond interactions between transcription factors and DNA, the approach reported in this study holds great promise for future research of noncovalent interactions by top-down MS, particularly for more complex biomolecular assemblies, whose interaction dynamics often remains unclear in solution.

## Supporting Information Available

Additional methods: Expression and purification of isotopically natural and depleted FOXO4-DBD, top-down data processing, electrophoretic mobility shift assay; Additional Figures: (Figure S1) FOXO4 sequence with both top-down and wild-type numbering, (Figure S2) ESI-MS spectra of desalted protein samples, (Figure S3) EMSA gel, (Figure S4) broadband ECD spectrum of IN-/ID-FOXO4-DBD, (Figure S5) quantified ions of IN-FOXO4-DBD, (Figure S6) zoom of [c4]^1+^ and [c5]^1+^ ions, (Figure S7) zoom of [z3]^1+^ and [z4]^1+^ ions, (Figure S8) quantified ions of ID-FOXO4-DBD, (Figure S9) zoom-in of ECD MSMS spectra of IN-/ID-FOXO4-DBD, (Figure S10) ab initio model of FOXO4-IRE with wild-type numbering, (Figure S11) bottom-up analysis of IN-FOXO4-DBD, (Figure S12) quantified extent of oxidation of W94 residue; Additional table: (Table S1) all modifications identified in bottom-up approach (PDF)
