# Supplementary material for: Isotopic Depletion Increases the Spatial Resolution of FPOP Top-Down Mass Spectrometry Analysis
Source: Anal Chem. 2024 Jan 16;96(4):1478–87. doi: 10.1021/acs.analchem.3c03759 (PMC10831798; doi:10.1021/acs.analchem.3c03759)
Supplement: Supplementary file 1 — ac3c03759_si_001.pdf [file ac3c03759_si_001.pdf]

Supporting information

Isotopic Depletion Increases the Spatial Resolution of FPOP Top-Down Mass Spectrometry Analysis.

Marek Polák[$,‡], Jiří Černý[†] and Petr Novák[*,$,‡]

$Institute of Microbiology of the Czech Academy of Sciences, 14200, Prague, Czech Republic
‡Department of Biochemistry, Faculty of Science, Charles University, 12800, Prague, Czech Republic
†Institute of Biotechnology of the Czech Academy of Sciences, 14200, Prague, Czech Republic

*Correspondence to: pnovak@biomed.cas.cz

Table of content:

1. Additional Materials and methods
    1.1.1 Expression of isotopically natural (IN) FOXO4-DBD
    1.1.2 Expression of isotopically depleted (ID) FOXO4-DBD
    1.1.3 Purification of ID- and IN- FOXO4-DBD
    1.1.4 Top-down data – processing
    1.1.5 Electrophoretic mobility shift assay (EMSA)

2. Additional Figures
    Figure S1: Sequence of FOXO4-DBD with denoted top-down numbering and wild-type numbering.
    Figure S2: ESI-FT-ICR MS spectra in a broadband mode of IN- and ID- FOXO4-DBD
    Figure S3: Electrophoretic mobility shift assay (EMSA) of IN- and ID-FOXO4-DBD.

Figure S4: Broadband m/z MSMS spectrum of isotopically natural and depleted FOXO4-DBD.
Figure S5: Sequence coverage of oxidized IN-FOXO4-DBD and the oxidation rates of fragment ions obtained ECD fragmentation of 3 singly oxidized charge states in multiCASI mode.
Figure S6: zoom of $[c4]^{1+}$ and $[c5]^{1+}$ fragment ion upon fragmentation of both IN- and ID-FOXO4-DBD.
Figure S7: zoom of $[z3]^{1+}$ and $[z4]^{1+}$ fragment ion upon fragmentation of both IN- and ID-FOXO4-DBD.
Figure S8: Sequence coverage of oxidized ID-FOXO4-DBD and the oxidation rates of fragment ions obtained ECD fragmentation of 3 singly oxidized charge states in multiCASI mode.
Figure S9: zoom in of MSMS spectra of IN- and ID-FOXO4-DBD displaying the improvements of isotopic depletion in top-down technology.
Figure S10: An *ab-initio* structural model FOXO4-DBD-IRE with denoted regions/residues that were observed as oxidized and numbered according wild-type FOXO4-DBD.
Figure S11: Digestion map of FOXO4-DBD, quantified residues detected in bottom-up analysis.
Figure S12: Quantified extent of oxidation of W173 residue by both top-down and bottom-up approach.
3. Additional Tables
Table S1: List of unambiguously identified modifications using bottom-up approach
4. References

1. Additional Materials and Methods

1.1 Expression of isotopically natural (IN) FOXO4-DBD

The pET-28(+) plasmid (Generey, China), containing the gene of DNA-binding domain of FOXO4 transcription factor (82-186), was used to transformed *E. coli* BL21-CodonPlus (DE3)-RIPL (Agilent Technologies, USA) and to express an N-terminal His_6-tagged recombinant protein. The bacteria were cultivated in Terrific Broth (TB) medium at 37 °C until OD_{600} = 0.9, the expression was induced by addition of 0.5mM isopropyl-β-D-thiogalactopyranoside (IPTG), continued by incubation at 18 °C for more 16 hours. The bacteria were harvested by centrifugation (8000 × *g*, 15 min, 4 °C) and stored at -80 °C for further use.

1.2 Expression of isotopically depleted (ID) FOXO4-DBD

Isotopically depleted protein was expressed in M9 minimal medium containing $CaCl_2$ (0.1mM), $MgSO_4$ (2mM), $FeCl_3$ (5µM), Thiamine hydrochloride (vitamin B1, 3mM), Biotin (vitamin B7, 3mM), Basal Medium Eagle vitamins (BME, 1× concentrated), Trace metals (1× concentrated), D-glucose (99.9 % of ^{12}C, 2 g/L) and NH_4SO_4 (99.99 % of ^{14}N, 2 g/L). A 50 µl of starter culture, growth in TB medium, was centrifuged (5000 × *g*, 5 min). TB-based supernatant was discarded, pellet was resuspended and transferred into 10 ml of fresh M9 medium, followed by incubation overnight at 37 °C to create M9 starter preculture. A one liter of M9 media was inoculated by the overnight preculture (500:1, V:V) and bacteria were cultivated at 37 °C until OD_{600} = 0.6; the expression was induced by addition of 0.5mM (IPTG). Incubation was continued for 12 hours at 18 °C. The bacteria were harvested from media by centrifugation (8000 × *g*, 15 min, 4 °C) and stored at -80 °C for further use.

1.3 Purification of ID- and IN- FOXO4-DBD

Bacterial pellets were resuspended in 20mM HEPES, 0.5M NaCl, 20mM imidazole, pH 7.4 buffer containing protease inhibitor cocktail, DNAse (300 U), RNAse (300U), and disintegrated by ultrasound on ice bath. The soluble fraction was separated by centrifugation (70 000 × *g*, 60 min, 4 °C), filtered using a 0.22 µm syringe filters (Millipore, Merck, USA). Supernatant was loaded on Ni^{2+}-charged Bio-Scale Mini Nuvia IMAC column (5 ml; Bio-Rad Laboratories, USA) already equilibrated in the 20mM HEPES buffer. Once the resin was washed with the HEPES buffer, the protein was eluted by linear gradient of 20-500 mM imidazole. Fractions corresponding FOXO4-DBD were dialyzed against 20mM HEPES, 50mM NaCl, pH 7.4 and N-terminal His_6-tag cleavage was done during dialysis with TEV protease[1] (1:100, m:m), overnight at 4 °C. ESI-FTICR MS analysis was carried out to verify the cleavage efficacy. The His_6-tagged TEV protease was separated from FOXO4-DBD by affinity chromatography on Bio-Scale Mini Nuvia IMAC column (1 ml; Bio-Rad Laboratories, USA), and flow-through fraction was loaded on EconoFit High S column (5 ml; Bio-Rad Laboratories, USA) to perform cation-exchange chromatography. Protein was further eluted by linear gradient of NaCl ranging 50-500 mM NaCl. The collected fractions were frozen in liquid nitrogen and stored at -80 °C until further use.

1.4 Top-down data – processing

Deconvolution of isotopically natural unmodified MS/MS spectra were done using SNAP algorithm method (Sophistical Numerical Annotation Procedure, Bruker Daltonics, USA). The quality factor threshold was set to 0.3 and S/N was set to 0. Maximum charge state was set as +14. Spectra were re-calibrated using a monoisotopic masses of a high-intensity fragments obtained by *in-silico* MS/MS fragmentation in GPMAW12.20 software[2], a 0.5ppm mass accuracy was achieved. This served to create a library of all possible c, z and y ions obtained from FOXO4-DBD sequence in MS2Links software[3]. Singly-oxidized MS/MS spectra were deconvoluted using FTMS algorithm, recalibrated as described above and exported as x/y coordinates (mass and corresponding intensity). The library of c/z/y ions and raw files were uploaded in our home-built software. Only singly-oxidized ions were searched in spectra with the ion-search mass accuracy under 3 ppm. Dataset were analyzed in a technical triplicate and quantified as already described[4,5]. Data are presented in a form ±SD and statistically analyzed by t-test in GraphPad Prism 8.0 software.

Data analysis of isotopically depleted (ID) FOXO4-DBD. An MS/MS spectra were re-calibrated in the same fashion as IN-version and exported using an FTMS algorithm. However, an *in-silico* library of all possible fragment ions and containing monoisotopic masses was created using an MS2Links software[3]. An MS/MS data of oxidized ID-version were then searched against the library of all possible fragment ions with the 3 ppm mass accuracy. Data were manually validated for each matched fragment ion. Dataset were analyzed in a technical triplicate and quantified as already described[4,5]. Data are presented in a form ±SD and statistically analyzed by t-test in GraphPad Prism 8.0 software.

1.5 Electrophoretic mobility shift assay (EMSA)

Samples containing 100 pmol of DNA were diluted into 150 mM ammonium acetate, pH 6.8 into a final volume of 10 µl. Gel Loading Dye (6× concentrated, New England Biolabs, USA) were added to each sample to the final concentration of 1× concentrated. Samples were loaded onto a 12% acrylamide gel and the electrophoretic assay run in the presence of TBE (Tris/Borate/EDTA) for ~45 min at 4 °C. The voltage was set to 150 V per gel. The gel was subsequently stained in GelRed® dye (Biotinum, USA) for 30 min and visualized using the CCD camera (Bio-Rad Laboratories, USA).

Additional Figures:

FOXO4 Top-down sequencing vs. wild-type numbering

Top-down numbering	1 **GS**GAVTGPRK	11 GGSRRNAWGN	21 QSYAELISQA	
Wild-type numbering	-2 **GS**GAVTGPRK	90 GGSRRNAWGN	100 QSYAELISQA	
	31 IESAPEKRLT	41 LAQIYEWMVR	51 TVPYFKDKGD	61 SNSSAGWKNS
	110 IESAPEKRLT	120 LAQIYEWMVR	130 TVPYFKDKGD	140 SNSSAGWKNS
	71 IRHNLSLHSK	81 FIKVHNEATG	91 KSSWWMLNPE	101 GGKSGKA
	150 IRHNLSLHSK	160 FIKVHNEATG	170 KSSWWMLNPE	180 GGKSGKA

Figure S1. The sequence of FOXO4 construct used in this study. The upper sequence represents the numbering arose from top-down fragmentation, thus 1-107. The lower sequence represents the FOXO4 wild-type numbering so the reader can convert information from fragment ions to the specific amino acid residue. The bolded sequence, GS, represents additional residues from multiple cloning site and they do not represent the wild-type sequence of FOXO4.

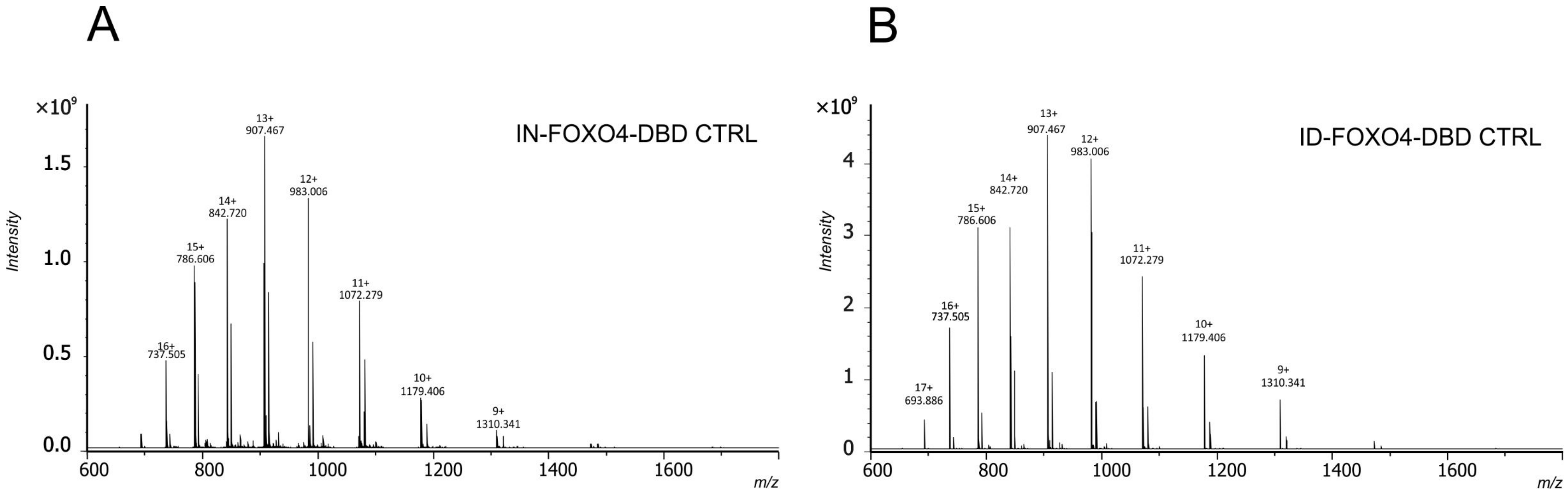

Figure S2. ESI-FT-ICR-MS analysis of isotopically natural (A) and isotopically depleted (B) unmodified FOXO4-DBD. The spectrum is zoomed to 600-1800 *m/z*, otherwise the data were acquired in a 207-2500 m/z broadband mode. The second population in spectra represents protein alongside a single phosphate adduct peak in spectrum.

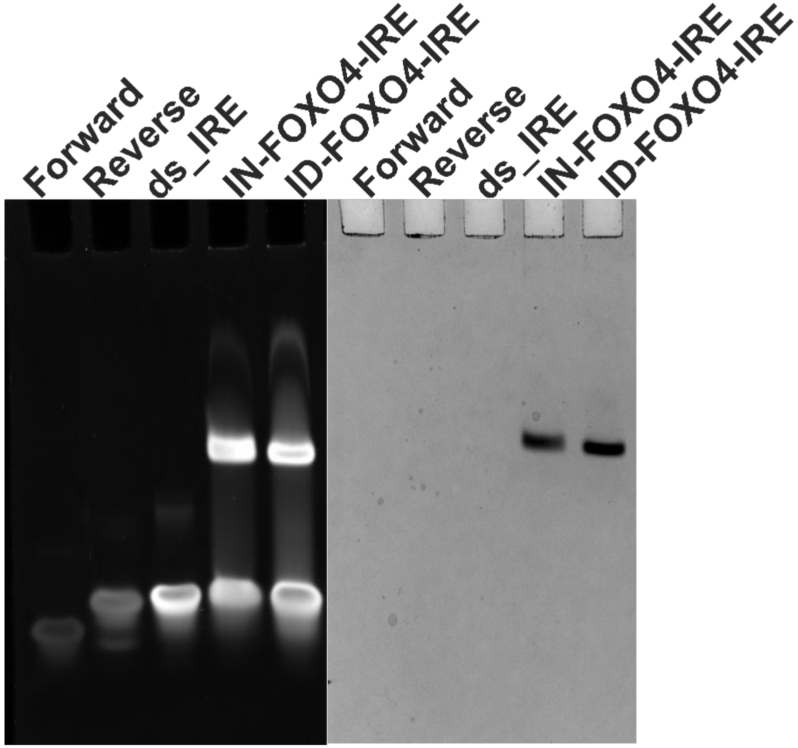

Figure S3. Electrophoretic mobility shift assay of both IN-FOXO4•dsIRE and ID-FOXO4•dsIRE complex stained by GelRed® (left panel) for nucleic acid and Coomassie Brilliant Blue for protein (right panel). The presence of both IN-FOXO4-DBD and ID-FOXO4-DBD resulted in mobility shift compared to the duplex IRE alone.

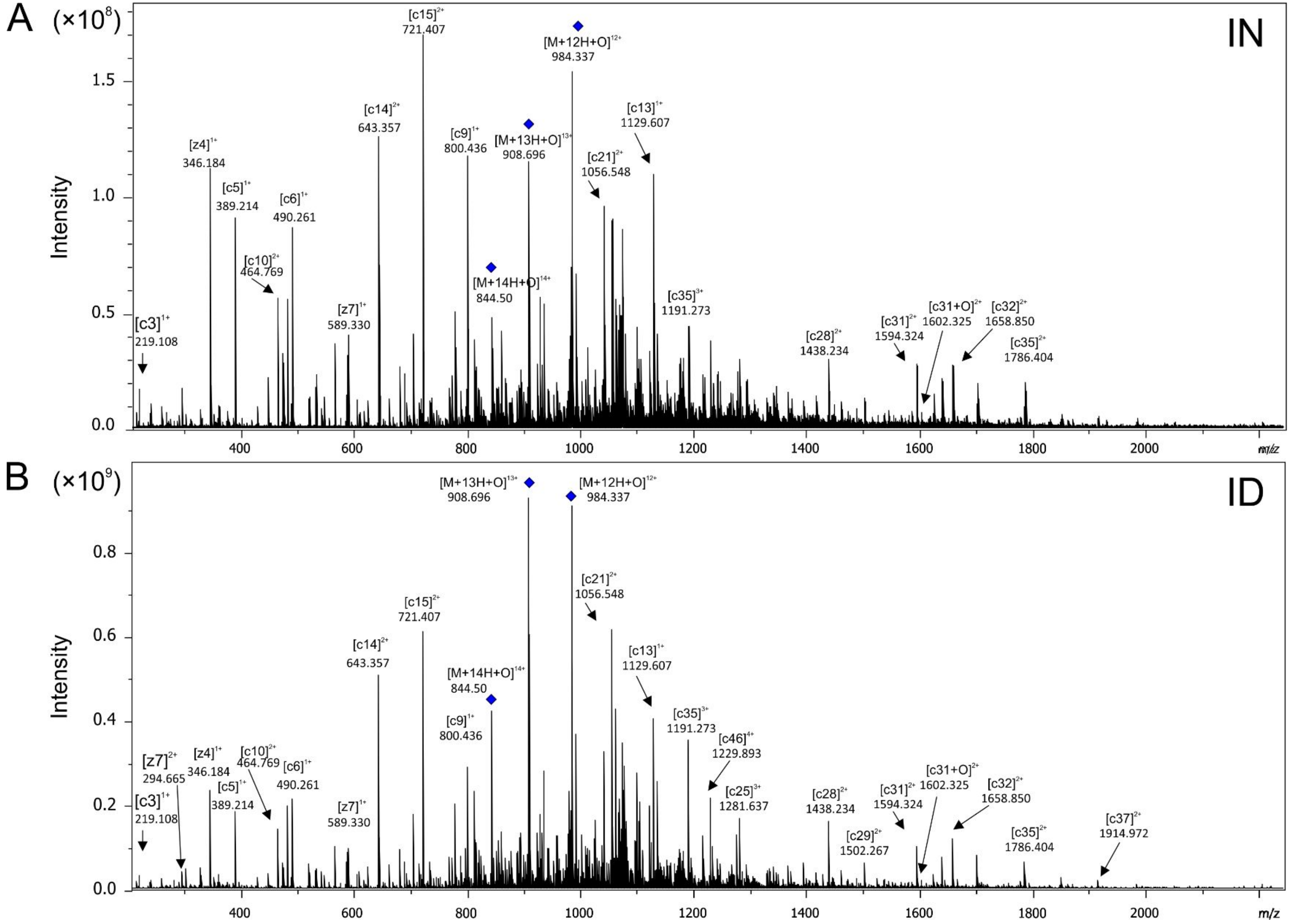

Figure S4. A. Broadband *m/z* MSMS spectrum upon fragmentation of isotopically natural FOXO4-DBD. B. Broadband *m/z* MSMS spectrum upon fragmentation of Isotopically depleted FOXO4-DBD. Fragment nomenclature and their monoisotopic masses are indicated upon the abundant fragment ions. The singly oxidized precursor of 14+, 13+ and 12+ charge state is denoted by blue diamond symbol.

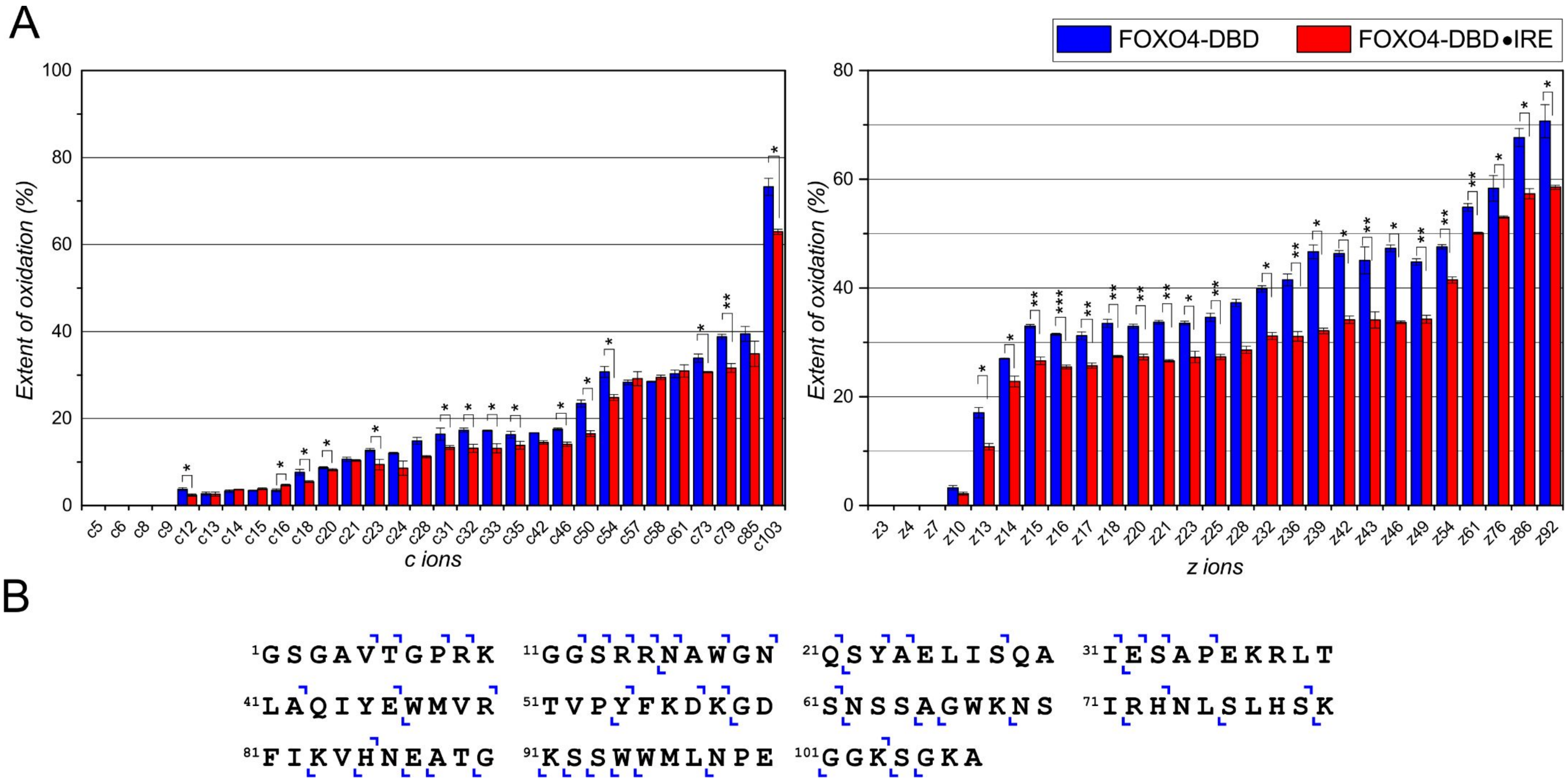

Figure S5. A. Extent of oxidation of fragment c ions (top panel) and z ions (bottom panel) of isotopically natural FOXO4-DBD without (blue colored ions) and with IRE-binding element (red colored ions). B. Sequence of FOXO4-DBD with the denoted fragment ions that were quantified. Note that the first 2 residues (GS) originate from bacterial express vector and thus do not represent the wild-type sequence of FOXO4 protein.

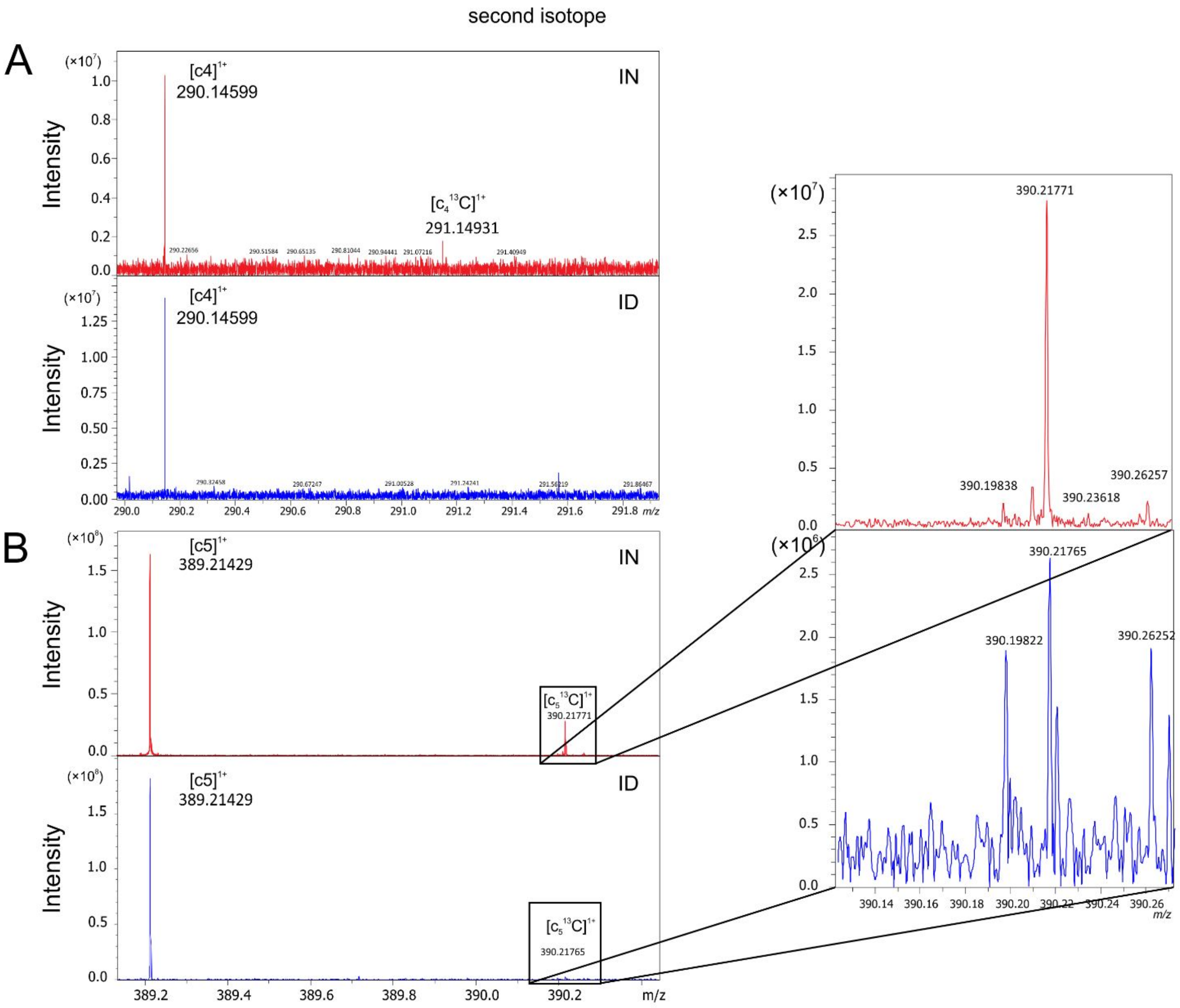

Figure S6. A. The zoom on a $[c4]^{1+}$ fragment ion appeared at a monoisotopic mass 290.14599 amu. Upon fragmentation of isotopically natural sample, the ion appears bearing two distinguished isotopic peaks (red spectrum) but missing the second isotopic peak upon fragmentation of isotopically depleted sample (blue spectrum). B. The zoom of $[c5]^{1+}$ fragment ion appears at a monoisotopic mass 389.21429 amu. Here the inset shows a region between 390.12 – 390.27 amu where the second isotope peak is visible in both isotopically natural and depleted MSMS spectra.

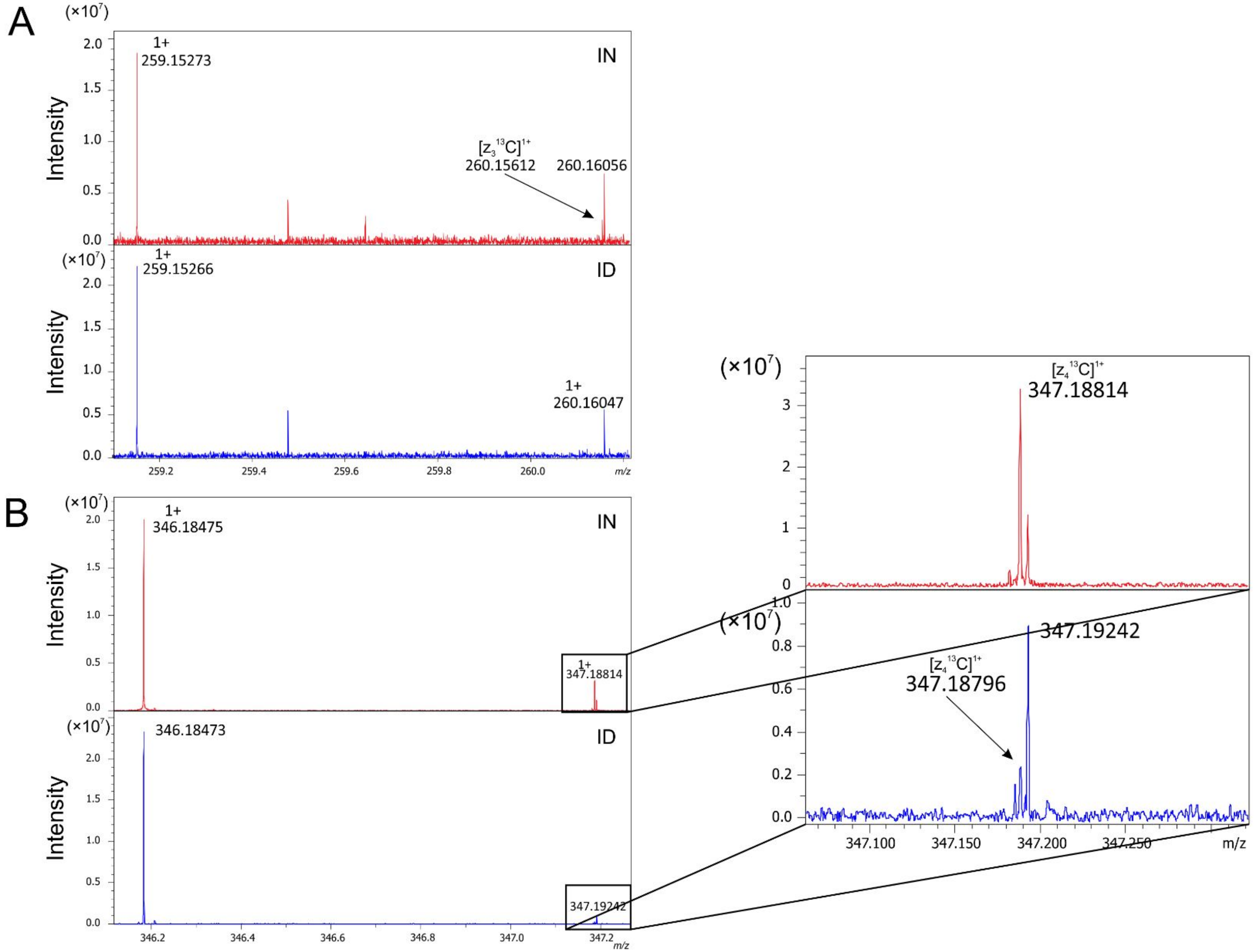

Figure S7. A. The zoom on a $[z3]^{1+}$ fragment ion appeared at a mass 259.15266 amu. Upon fragmentation of isotopically natural sample, the ion appears bearing two distinguished isotopic peaks (red spectrum) but missing the second isotopic peak upon fragmentation of isotopically depleted sample (blue spectrum). B. The zoom of $[z4]^{1+}$ fragment ion appears at a monoisotopic mass 346.18473 amu. Here the inset shows a region between 347.020 – 347.70 amu where the second isotope peak is visible in both isotopically natural and depleted MSMS spectra.

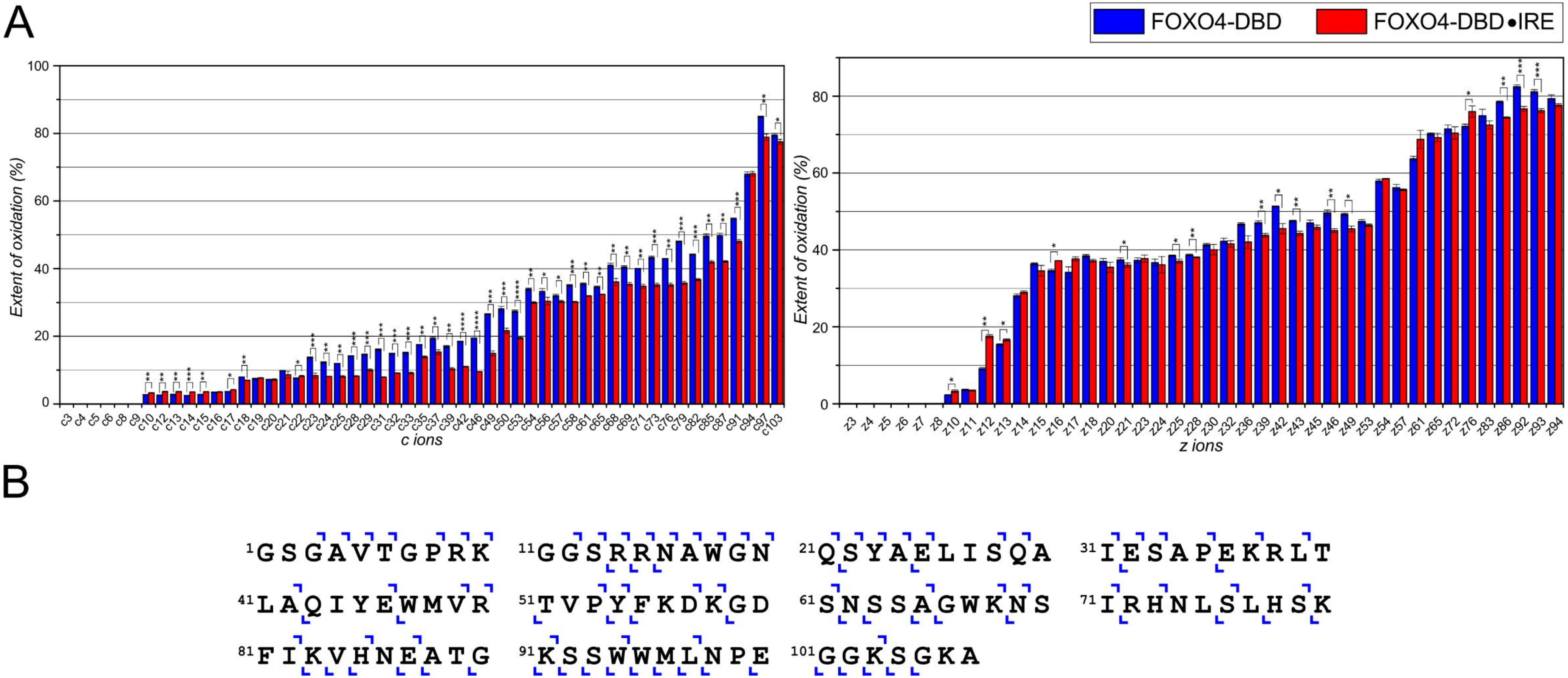

Figure S8. A. The extent of oxidation of fragment c ions (top panel) and z ions (bottom panel) of isotopically depleted FOXO4-DBD without (blue colored ions) and with IRE-binding element (red colored ions). B. Sequence of FOXO4-DBD with the denoted fragment ions that were quantified. Note that the first 2 residues (GS) originate from bacterial express vector and thus do not represent the wild-type sequence of FOXO4 protein.

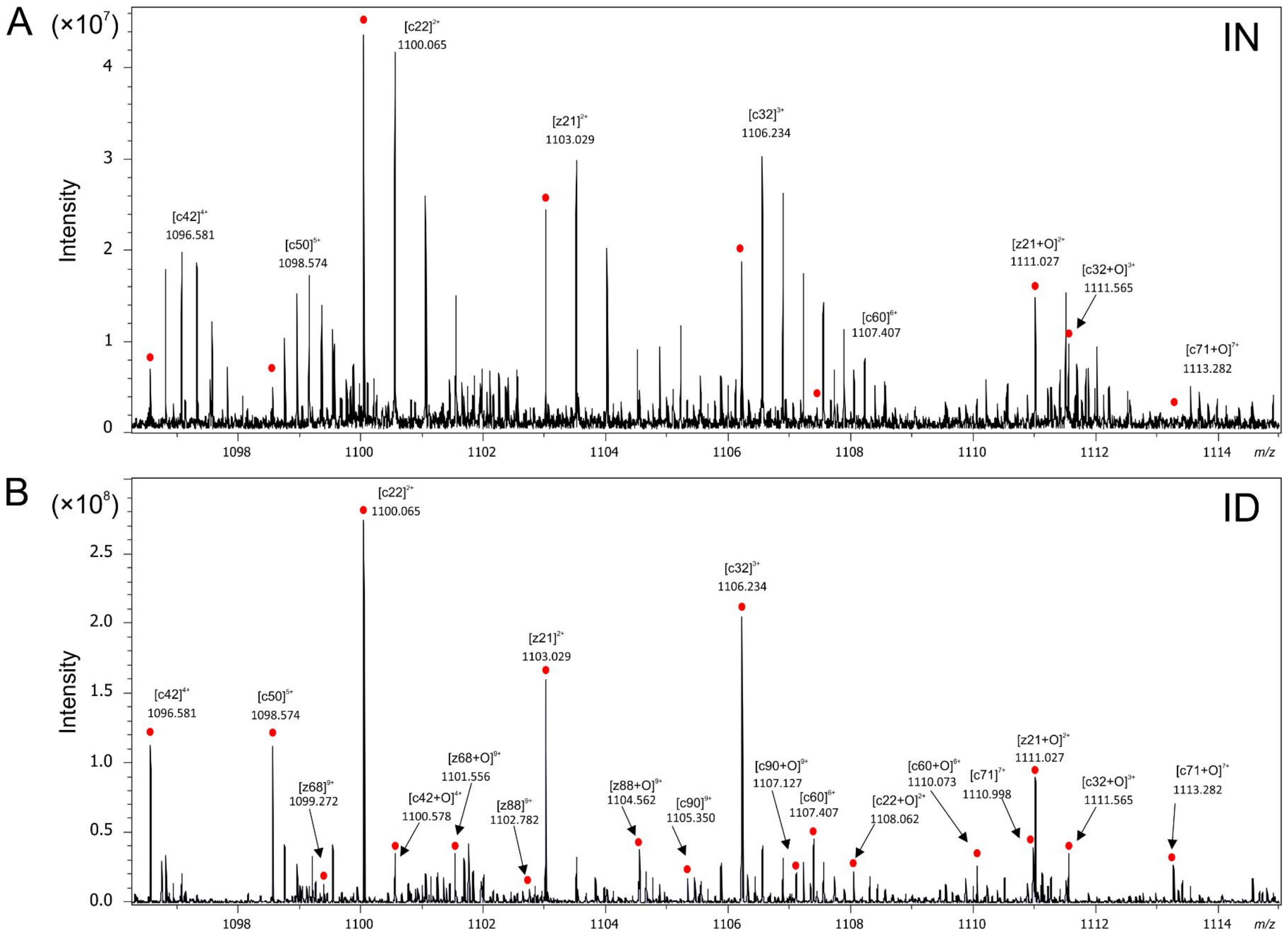

Figure S9. A. Zoom-in on MSMS spectra between 1096 – 1115 amu upon fragmentation of isotopically natural (A) and isotopically depleted (B) sample. Fragment ions or their oxidized forms are denoted above the fragments with their monoisotopic masses. The red dot represents the position of monoisotopic peaks of particular fragment ions.

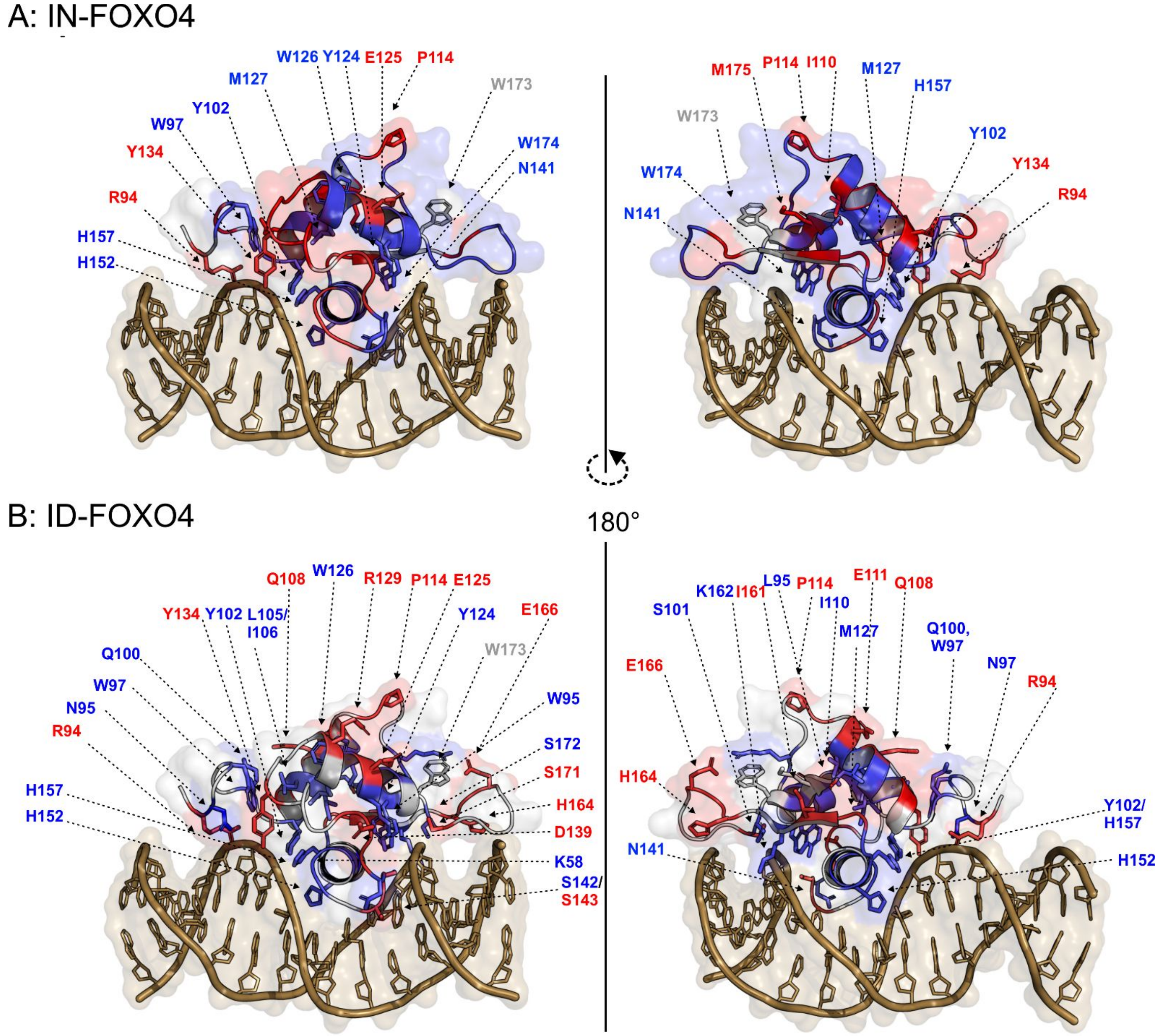

Figure S10. An *in-silico* structural model of FOXO4-DBD•IRE (PDB template 3L2C)22 with the highlighted differently oxidized regions/residues detected by both top-down analyses for natural version (A) or depleted version (B) of FOXO4-DBD. The individual residues detected in either bottom-up approach or deduced from top-down were highlighted into the model and colored. Blue – regions/residues detected as more modified in apo form, red – regions/residues detected as more modified in holo form. The residues are numbered according to the FOXO4 wild-type sequence.

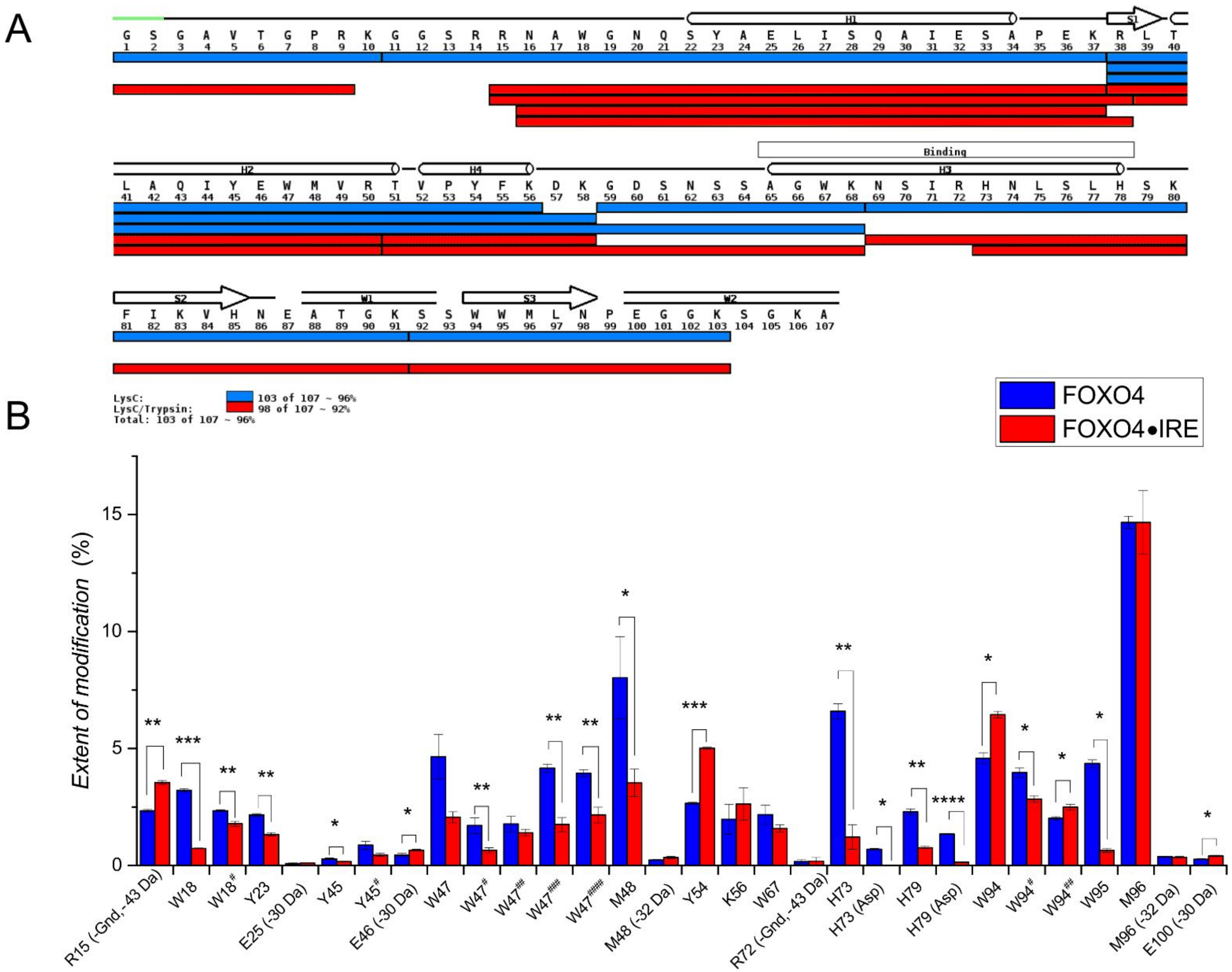

Figure S11. A. Sequence coverage map of peptides created by digestion using LysC (colored in blue) or combination Try/LysC (colored in red). Combining both proteases gives rise to the sequence coverage of 96 %. The green line covering the first two amino acids represents the multiple cloning site and thus do not represent the native FOXO4 sequence. B. Quantified extents of modification or residues detected in LC-MS/MS analysis. Blue – extent of modification or residues in reaction without the IRE, red – extents of modification of residues in FPOP reaction with the IRE.

Modification different than +15.9949 Da (addition of oxygen) are indicated in brackets. Positional isomers of particular residue are denoted with hashtag(s). The results of the t-test are indicated by a number of asterisks above the histograms. T-test legend: * (P ≤ 0.05), ** (P ≤ 0.01), *** (P ≤ 0.001), **** (P ≤ 0.0001).

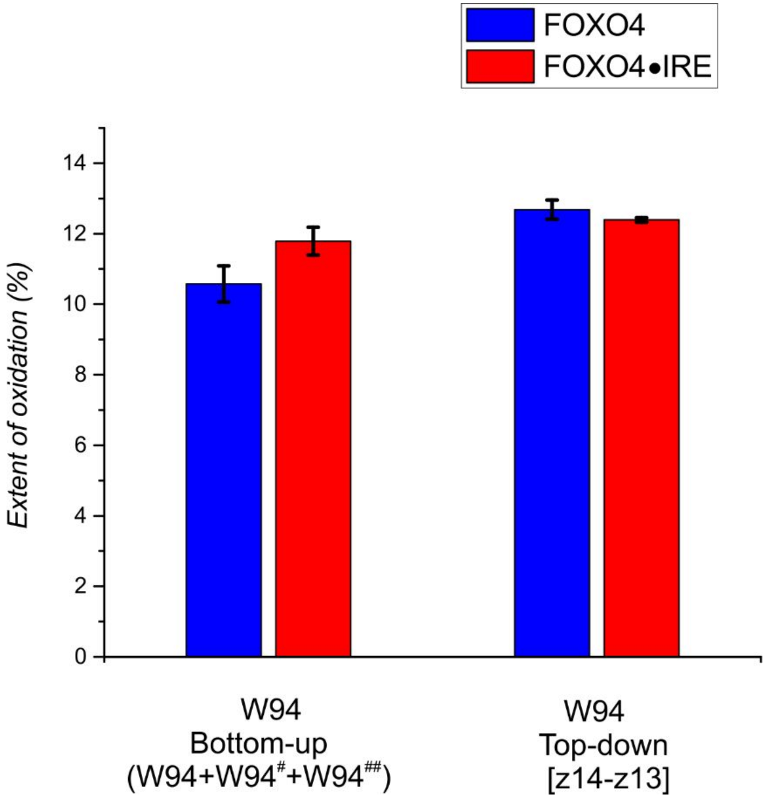

Figure S12. A. The extent of oxidation of W94 residue (native numbering W173) quantified by both bottom-up and top-down approaches. The values for bottom-up approach were created by summing all separated isomers of W94 residue (Figure S11 – W94+W94#+W94##) observed in LC-MS analysis (Table S1). The values for the top-down approach were created by subtracting the extent of oxidation of [z13] fragment ion from [z14] fragment ion (Figure S8).

2. *Additional Tables*

Table S1: List of unambiguously identified modifications using bottom-up approach (Table follows on the second page).

			FOXO4-DBD		FOXO4-DBD + IRE			
			Extent of modification (%)		Extent of modification (%)		t-test	
Peptide	Residue	Residue (FOXO4 wild-type numbering)	AVG	SD	AVG	SD	p value	//*
11-37	R15 (-Gnd)	R94 (-Gnd)	2.341925	0.058421	3.548012968	0.082637	0.0029	**
16-37	W18	W97	3.216409	0.067029	0.733954091	0.019567	0.0001	***
16-37	W18#	W97#	2.34441	0.040033	1.783368201	0.101798	0.0058	**
16-37	Y23	Y102	2.165392	0.051686	1.326937381	0.061069	0.0015	**
16-37	W18, Y23	W97, Y102	0.339194	0.017458	0.221277062	0.012393	0.0007	***
16-37	W18+2O	W97+2O	0.366837	0.016883	0.23074932	0.004641	0.0052	**
16-37	W18+3O	W97+3O	0.457929	0.066702	0.4994677	0.057659	0.5856	ns
16-37	E25 (-30 Da)	E104 (-30 Da)	0.085247	0.024592	0.09951372	0.003525	0.378	ns
38-50	Y45	Y124	0.2776	0.039899	0.165848265	0.01144	0.0459	*
38-50	Y45#	Y124#	0.868854	0.172054	0.451216492	0.066527	0.0765	ns
39-50	E46 (-30 Da)	E125 (-30 Da)	0.446972	0.070613	0.655921444	0.037991	0.0178	*
38-50	W47	W126	4.650507	0.949005	2.061088569	0.238085	0.0508	ns
38-50	W47#	W126#	1.706221	0.331944	0.651961894	0.114272	0.0498	*
38-50	W47##	W126##	1.774002	0.328815	1.404999474	0.145729	0.2165	ns
38-50	W47###	W126###	4.157	0.173299	1.751781659	0.301743	0.001	**
39-50	W47####	W126####	3.947002	0.145448	2.159376248	0.333856	0.0044	**
38-50	M48	M127	8.026071	1.759255	3.533605988	0.585778	0.0043	**
39-50	M48 (-32Da)	M127 (-32Da)	0.235104	0.029214	0.342165981	0.045572	0.0428	*
38-50	W47, M48	W126, M127	0.629223	0.163988	0.295521766	0.064416	0.0573	ns
38-50	W47, Y45	W126, Y124	0.202829	0.070047	0.115485725	0.011568	0.1724	ns
38-50	Y45+2O	Y124+2O	0.117105	0.023628	0.096671119	0.016052	0.3731	ns
38-50	W47+2O	W126+2O	4.154115	0.162319	2.215624199	0.400628	0.0233	*
39-50	M48+2O	M127+2O	0.431484	0.067985	0.247270279	0.039627	0.0199	*
39-50	W47+2O	W126+2O	2.193539	0.054078	1.6790892	0.205913	0.0546	ns
51-58	Y54	Y133	2.655439	0.051893	5.016910665	0.036263	0.0001	***
51-58	K56	K135	1.975931	0.645407	2.63119033	0.687401	0.2749	ns
59-68	W67	W146	2.173224	0.412564	1.581963137	0.157143	0.1207	ns
68-80	R72 (-Gnd)	R151 (-Gnd)	0.16107	0.083786	0.189260307	0.153883	0.5602	ns
68-80	H73	H152	6.586604	0.330228	1.219477301	0.521255	0.0083	**
68-80	H73 (Asp)	H152 (Asp)	0.690993	0.040103	0	0	0.0018	**
73-80	H78	H157	2.298201	0.119416	0.764359127	0.047808	0.0037	**
73-80	H78 (Asp)	H157 (Asp)	1.345366	0.017791	0.140866284	0.030134	<0.0001	****
92-103	W94	W173	4.583312	0.23356	6.450574666	0.142178	0.0123	*
92-103	W94#	W173#	3.97266	0.204084	2.840809869	0.134489	0.0284	*
92-103	W94##	W173##	2.019902	0.072183	2.494634462	0.119118	0.0428	**
92-103	W95	W174	4.357071	0.157088	0.641490947	0.080133	0.0007	***

92-103	M96	M175	14.66789	0.260322	14.66681738	1.353177	0.9992	ns
92-103	M94 (-32 Da)	M175 (-32 Da)	0.371602	0.018925	0.35252609	0.039585	0.5998	ns
92-103	W94, M96	W173, M175	2.538074	0.098345	2.854126228	0.290708	0.2938	ns
92-103	W94, W95	W173, W174	2.062432	0.353258	1.495295826	0.116485	0.086	ns
92-103	W95, M96	W174, M175	0.48903	0.022086	0.228779243	0.011578	0.0055	**
92-103	W94+2O	W173+2O	4.559321	0.16092	6.935200268	0.432187	0.0149	*
92-103	W95+2O	W174+2O	0.577663	0.040927	0.277815336	0.012977	0.0141	*
92-103	E100 (-30 Da)	E179 (-30 Da)	0.268716	0.019106	0.410026526	0.036237	0.0414	*

AVG – arithmetic average, SD – standard deviation, Extent – extent of modification (%). T-test legend: ns – not significant, * ($P \leq 0.05$), ** ($P \leq 0.01$), *** ($P \leq 0.001$), **** ($P \leq 0.0001$). * Negative numbering of residues refers to the residues originating from a bacterial vector.

3. References

(1) Tropea, J. E.; Cherry, S.; Waugh, D. S. Expression and Purification of Soluble His6-Tagged TEV Protease. *Methods Mol. Biol.* **2009**, *498*, 297–307. https://doi.org/10.1007/978-1-59745-196-3_19/COVER.

(2) Peri, S.; Steen, H.; Pandey, A. GPMAW – a Software Tool for Analyzing Proteins and Peptides. *Trends Biochem. Sci.* **2001**, *26* (11), 687–689. https://doi.org/10.1016/S0968-0004(01)01954-5.

(3) Kellersberger, K. A.; Yu, E.; Kruppa, G. H.; Young, M. M.; Fabris, D. Top-Down Characterization of Nucleic Acids Modified by Structural Probes Using High-Resolution Tandem Mass Spectrometry and Automated Data Interpretation. *Anal. Chem.* **2004**, *76* (9), 2438–2445. https://doi.org/10.1021/ac0355045.

(4) Polák, M.; Yassaghi, G.; Kavan, D.; Filandr, F.; Fiala, J.; Kukačka, Z.; Halada, P.; Loginov, D. S.; Novák, P. Utilization of Fast Photochemical Oxidation of Proteins and Both Bottom-up and Top-down Mass Spectrometry for Structural Characterization of a Transcription Factor–DsDNA Complex. *Anal. Chem.* **2022**, *94* (7), 3203–3210. https://doi.org/10.1021/acs.analchem.1c04746.

(5) Yassaghi, G.; Kukačka, Z.; Fiala, J.; Kavan, D.; Halada, P.; Volný, M.; Novák, P. Top-Down Detection of Oxidative Protein Footprinting by Collision-Induced Dissociation, Electron-Transfer Dissociation, and Electron-Capture Dissociation. *Anal. Chem.* **2022**, *94* (28), 9993–10002. https://doi.org/10.1021/ACS.ANALCHEM.1C05476.

(6) Boura, E.; Rezabkova, L.; Brynda, J.; Obsilova, V.; Obsil, T. Structure of the Human FOXO4-DBD–DNA Complex at 1.9 Å Resolution Reveals New Details of FOXO Binding to the DNA. *Acta Crystallogr. Sect. D Biol. Crystallogr.* **2010**, *66* (12), 1351–1357. https://doi.org/10.1107/S0907444910042228.
